# Diversity of Amyloid-beta Proteoforms in the Alzheimer’s Disease Brain

**DOI:** 10.1038/s41598-017-10422-x

**Published:** 2017-08-25

**Authors:** Norelle C. Wildburger, Thomas J. Esparza, Richard D. LeDuc, Ryan T. Fellers, Paul M. Thomas, Nigel J. Cairns, Neil L. Kelleher, Randall J. Bateman, David L. Brody

**Affiliations:** 1 Department of Neurology, Washington University School of Medicine, 660 South Euclid Avenue, St. Louis, MO 63110 United States; 2 Proteomics Center of Excellence, Northwestern University, Evanston, IL United States; 3 Department of Neurology, Knight Alzheimer’s Disease Research Center, 4488 Forest Park Pkwy, St. Louis, MO 63112 United States; 4 Department of Pathology and Immunology, 660 South Euclid Avenue, St. Louis, MO 63110 United States; 5 Department of Neurology, Hope Center for Neurological Disorders, 660 South Euclid Avenue, St. Louis, MO 63110 United States; 6 Department of Molecular Biosciences, Northwestern University, Evanston, IL United States; 7 Department of Chemistry, Northwestern University, Evanston, IL United States

## Abstract

Amyloid-beta (Aβ) plays a key role in the pathogenesis of Alzheimer’s disease (AD), but little is known about the proteoforms present in AD brain. We used high-resolution mass spectrometry to analyze intact Aβ from soluble aggregates and insoluble material in brains of six cases with severe dementia and pathologically confirmed AD. The soluble aggregates are especially relevant because they are believed to be the most toxic form of Aβ. We found a diversity of Aβ peptides, with 26 unique proteoforms including various N- and C-terminal truncations. N- and C-terminal truncations comprised 73% and 30%, respectively, of the total Aβ proteoforms detected. The Aβ proteoforms segregated between the soluble and more insoluble aggregates with N-terminal truncations predominating in the insoluble material and C- terminal truncations segregating into the soluble aggregates. In contrast, canonical Aβ comprised the minority of the identified proteoforms (15.3%) and did not distinguish between the soluble and more insoluble aggregates. The relative abundance of many truncated Aβ proteoforms did not correlate with post-mortem interval, suggesting they are not artefacts. This heterogeneity of Aβ proteoforms deepens our understanding of AD and offers many new avenues for investigation into pathological mechanisms of the disease, with implications for therapeutic development.

## Introduction

Aβ has been the major target for disease-modifying therapeutic development in AD, driven in large part by studies of Down syndrome and studies of autosomal dominant AD and sporadic, late-onset AD, which implicate increased production, decreased clearance, and Aβ aggregation^1, 2^. However, little is known about the proteoforms (*i*.*e*., all protein variants of a single gene including post-translational modifications and sequence variants)^3^ of Aβ in human AD brain. Previous studies have demonstrated some sequence heterogeneity and post-translational modifications (PTMs) of the Aβ peptide in amyloid-beta plaques^4–9^. Yet amyloid-beta plaques, one of the pathological hallmark of AD, correlate only moderately with dementia^10–12^. As a result, focus has shifted in recent years to the most toxic forms of Aβ, soluble aggregates previously termed ‘oligomers’ and other appellations^13^, as they demonstrate a strong correlation with dementia^14–19^.

**Figure 1:**
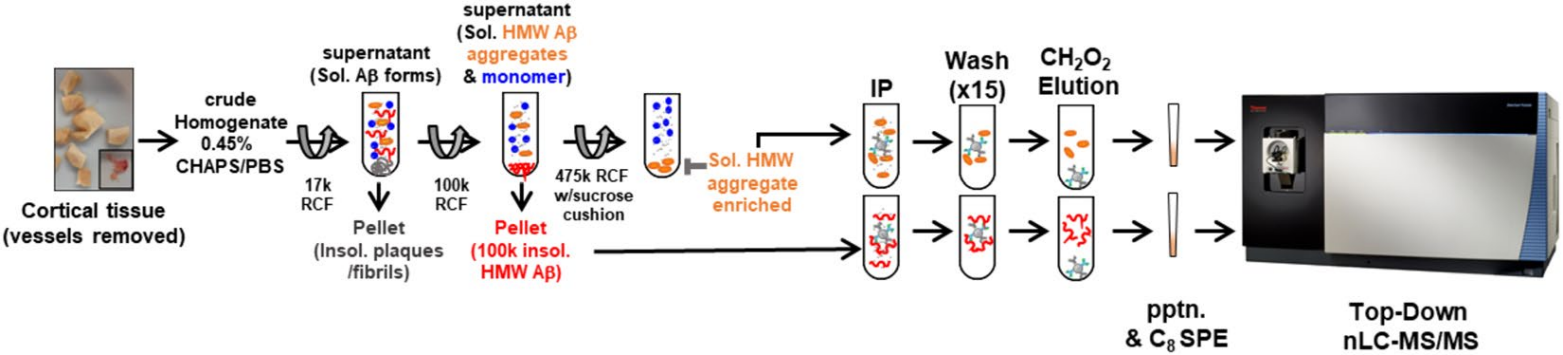
Isolation and comparison of Aβ proteoforms from human AD brain. Workflow for isolation of endogenous Aβ proteoforms. Cortical tissue was dounce homogenized with sub-critical micelle concentration of CHAPS followed by differential ultracentrifugation, anti-Aβ dual antibody immunoprecipitation, and elution in neat formic acid. Immunoprecipitated Aβ proteoforms in the HMW soluble aggregates fractions and more insoluble fractions were precipitated and purified from undigested non-Aβ proteins using C_8_ TopTips and analyzed by nLC-MS/MS. RCF, relative centrifugal force; Sol., soluble; LMW, low molecular weight; HMW, high molecular weight; IP, immunoprecipitation; pptn., precipitation; SPE, solid phase extraction.

Toward this end, we used high-resolution mass spectrometry to obtain highly accurate measurements and peptide-sequencing information of intact, undigested Aβ from soluble and insoluble Aβ aggregates isolated from human AD brain (Table 1). Soluble Aβ aggregates were isolated with our fully quantitative, sensitive, specific, and efficient purification strategy^20^ (Fig. 1). For comparison, more insoluble Aβ aggregates were analyzed from the same brain samples. Analysis of undigested (*i*.*e*., top-down proteomics) peptides by nano-liquid chromatography tandem mass spectrometry (nLC-MS/MS) allows unambiguous identification of Aβ and fully characterizes the composition of individual proteoforms. The advantages of nLC-MS/MS over other techniques like matrix-assisted laser desorption/ionization (MALDI) include: *i*) chromatographic separation of various proteoforms before mass spectrometry analysis, which offers better sample separation to effectively reduce sample complexity and improve resolution, *ii*) higher mass accuracy for the intact proteoforms, and *iii*) identification of fragment ions from direct *de novo* sequencing. Purification of soluble Aβ aggregates from frozen human AD brain by differential ultracentrifugation and immunoprecipitation resulted in ~10,000-fold enrichment and ~60% recovery of starting material^20^. Even so, Aβ comprised approximately 0.1% of the total protein content of the soluble high molecular weight (HMW) aggregates (Supplementary Table 1), requiring further enrichment before mass spectrometry analysis. We achieved substantial additional separation of Aβ from the other, as-yet-uncharacterized protein components of the soluble Aβ aggregates through an optimized C_8_ solid phase extraction protocol adaptable to varying amounts of total Aβ input (Supplementary Fig. 1; Supplementary Table 2). Using these methods, we analyzed Aβ extracted from brain tissue of six cases of AD each of whom had clinically severe AD dementia at expiration in an untargeted mass spectrometry approach (Supplementary Figs 2–4).

**Table 1:** Characteristics of Human Brain Frontal Cortex Samples.

Pt No.	Status	Age, yr	PMI, hr	Gender
1	CDR 3	73	12	male
2	CDR 3	83	6.3	female
3	CDR 3	92	18	female
4	CDR 3	86	15.5	female
5	CDR 3	89	6	female
6	CDR 3	82	10.75	male

**Figure 2:**
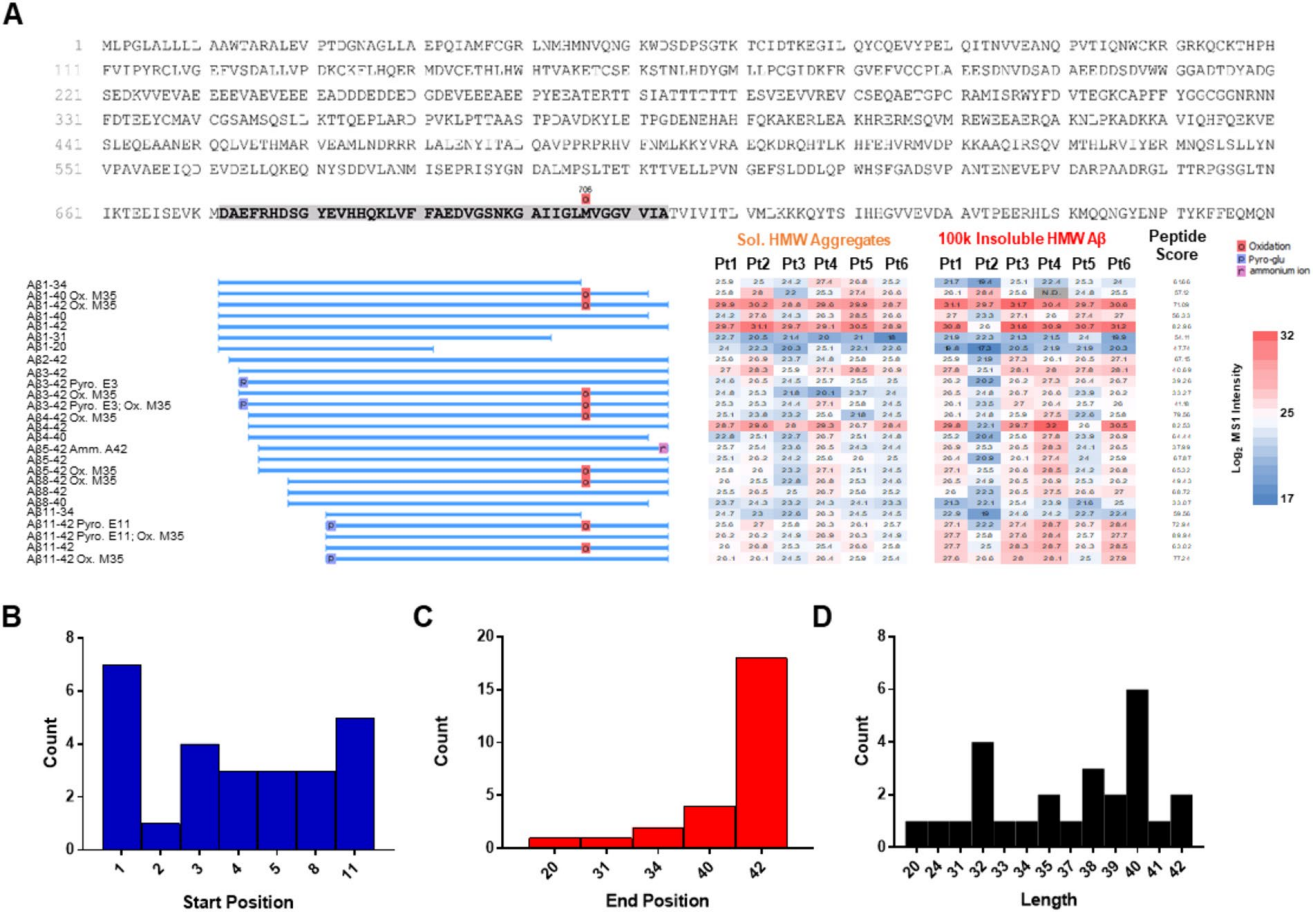
Heterogeneity of Aβ proteoforms in the human Alzheimer’s disease brain. (**A**) *Top:* full protein sequence of amyloid precursor protein with Aβ1–42 sequence highlighted (*grey*). *Left:* Identified Aβ proteoform sequence names, with blue bars representing the alignment with canonical Aβ. *Right:* Heatmap and quantification of the log_2_ relative intensity of each Aβ proteoform for each of the six severe Alzheimer’s disease brain samples in the soluble aggregate and more insoluble fractions. *Far right:* Peptide score, indicated confidence of peptide identification^38^. (**B**) Frequency of Aβ proteoform amino acid start positions identified in CDR3 cohort; 1 = D, aspartic acid. (**C**) Frequency of Aβ proteoform amino acid end positions identified in CDR3 cohort; 42 = A, alanine. (**D**) Frequency of Aβ proteoform lengths identified in CDR3 cohort. Pt., Participant; HMW, high molecular weight; N.D., not detected; Ox., Oxidation; Pyro., Pyro-glutamate; Amm., ammonium ion.

**Figure 3:**
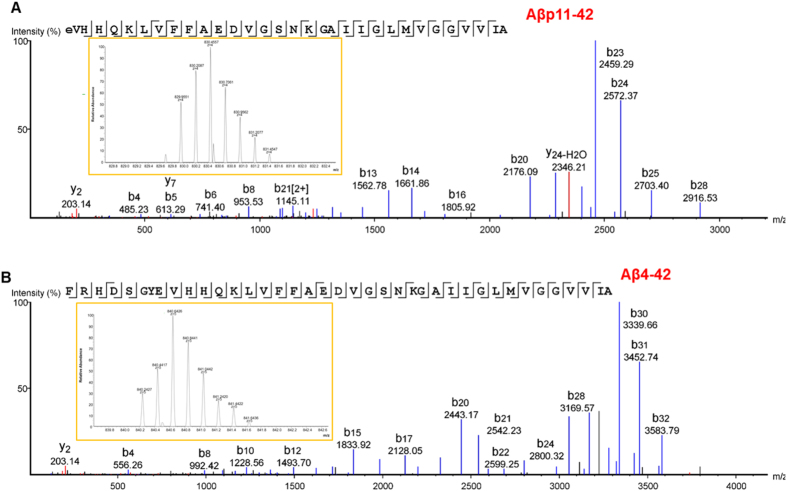
Mass spectrometry of Aβ proteoforms from human AD brain. (**A**) nano-Liquid chromatography-tandem mass spectrometry (nLC-MS/MS) spectrum for undigested, full-length Aβp11–42. Each peak represents an ion fragmented from Aβp11–42, with peaks labeled ‘*b*’ representing N-terminal fragment ions and peaks labeled ‘*y*’ representing C-terminal fragment ions. The numbers indicate measured mass-charge ratio (*m*/*z*). The single letter amino acid code across the top indicates the *de novo* sequence identified by mass spectrometry, which matches the amyloid precursor protein sequence corresponding to Aβp11–42. The line breaks between amino acids indicate a cleavage of the amide bond between two adjacent amino acids producing fragment ions. The lines below each amino acid indicate a detected ‘*b*’ ion, and lines above indicate a detected ‘*y*’ ion. *Inset:* isotopic envelope for the +4 charged, full-length Aβp11–42; the peaks are spaced 0.25 daltons apart at *z* = +4 because the naturally occurring isotopes (e.g. ^13^C and ^15^N) differ by 1 dalton. For the +4 ion, the observed *m/z* was 829.9551 (theoretical *m*/*z* = 829.9523), which was 3.4 parts-per-million (ppm) error from the theoretical mass of Aβp11–42. **(B**) Spectrum for full length Aβ4–42. For the +5 ion, the observed *m/z* was 840.2427 (theoretical *m*/*z* = 840.2399), which was 3.3 ppm error from the theoretical mass of Aβ4–42.

**Figure 4:**
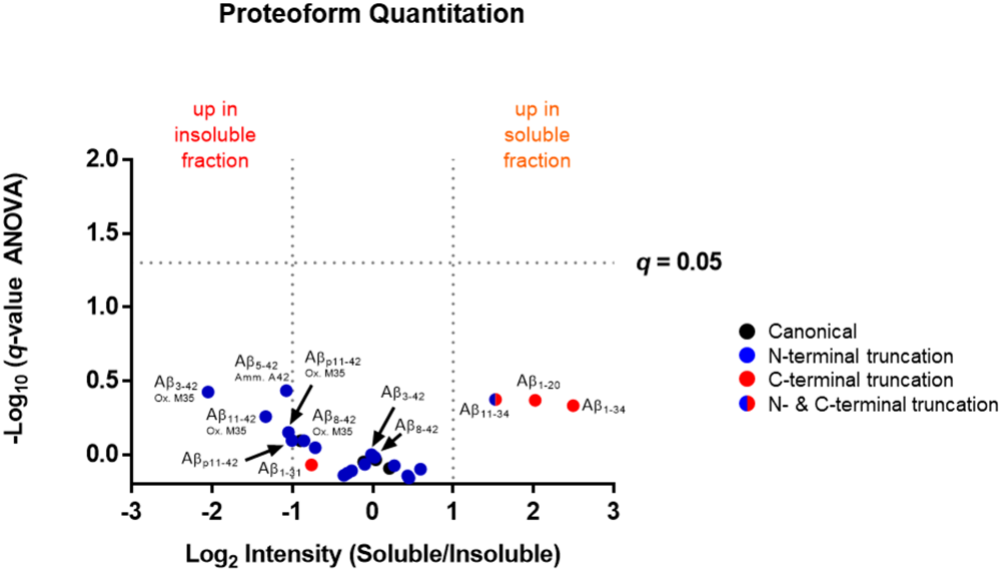
Determination of significant differentially expressed Aβ proteoforms. Logarithmic ratios of soluble *versus* insoluble fractions are plotted against the negative logarithmic *q*-values (*q* is a false discovery rate corrected p-value, where *q* ≤ 0.05 is considered statistically significant; above horizontal line) of the hierarchical linear model with replicates nested within participants and participants treated as random effects to test the fixed effect difference between soluble and insoluble aggregates. Vertical lines designate the logarithmic ratio indicating ±2-fold change. All Aβ proteoforms are listed in Supplementary Table 3 with their respective *q*-values and log_2_ fold change. Ox., Oxidation; Amm, ammonium ion; M, methionine; A, alanine.

We identified 26 Aβ proteoforms in AD brain (Fig. 2). The proteoforms were identified with a high degree of mass accuracy, as were fragment ions that allowed unambiguous determination of their primary structures (Fig. 3, Supplementary Fig. 5). Contained within our dataset were numerous forms of Aβ with N-terminal truncations (Aβx-40/42), C-terminal truncations (Aβ1-x), and in one case (Aβ11-34), both N- and C-terminal truncations (Fig. 2). The canonical forms of Aβ traditionally studied, Aβ1–42 and Aβ1–40, were present throughout all participants in all fractions. Aβ1–42, in both its oxidized and un-oxidized forms had one of the highest relative abundances in the mass spectrometry datasets. Aβ1–40 was also abundant, but with more variability amongst participants in both fractions, particularly in its oxidized form. Interestingly, no Aβ1–38, or any 38- or 37-terminating proteoforms, were identified in our dataset even though this species is commonly detected in cerebrospinal fluid (CSF)^21, 22^.

The N-terminal truncations identified include Aβ2-x, Aβ3-x, Aβ4-x, Aβ5-x, Aβ8-x, and Aβ11-x. C-terminal truncated forms of Aβ include Aβx-20, Aβx-31, and Aβx-34 (Fig. 2A). The most frequent start and end positions were at 1 (D; aspartic acid) and 42 (A; alanine), respectively (Fig. 2B,C), like canonical Aβ1–42. However closer inspection revealed that the most frequent proteoform length was 40 amino acids followed by 32 (Fig. 2D) and not a full 42 amino acid sequence. Only 4 out of 26 proteoforms were full-length (15.3%). The heterogeneity in sequence length can be largely attributed to the N-terminal heterogeneity (Fig. 2B) as 19 out of the 26 (73%) proteoforms contained N-terminal truncations as has been previously observed^22, 23^. While less frequent by comparison, 8 out of 26 (30%) proteoforms contained C-terminal truncations. Overall, the majority of proteoform sequences terminated at alanine 42 (18 out of 26, 69.2%; Fig. 2C) compared to 9.5% terminating in valine 40 (4 out of 26) in keeping with previous studies demonstrating that Aβx-42 aggregation rates were higher in amyloidosis^24^. Traditionally used Aβ1–40, Aβ1–42, or Aβ1-x (*i*.*e*., canonical N-terminus) enzyme-linked immunosorbent assays (ELISAs) would not detect the majority of these forms, which fits well with our previous report that much of the Aβ measured by ELISA in human brain interstitial fluid is neither Aβ1–40 nor Aβ1–42^21^.

The post-translationally modified forms of Aβ comprised N-terminal pyro-glutamate, oxidation of methionine, and in one instance an ammonium ion adduct at the C-terminus, which to our knowledge has not been previously reported. No reagents in our isolation protocol contained ammonia. Many of the other forms, including pyro-glutamate modification of amino acid 11 and truncated Aβ4–42, were of high relative abundance similar to canonical Aβ1–42. One caveat to cross-proteoform comparison is that the relative abundance in the mass spectrometry datasets is largely dependent on the ionization efficiency of various Aβ proteoforms. The ionization efficiency can be altered by sequence truncations and/or PTMs. Thus, the most reliable quantitative comparisons can only be made for a given proteoform between patients or fractions in this study.

Differential mass spectrometry (dMS) analysis revealed no statistically significant differences in Aβ proteoform abundance of the 26 Aβ proteoforms identified using two independent platforms, between soluble aggregates and the more insoluble material (Fig. 4; Supplementary Table 3)^25^. However, it did uncover trends in regard to the types of proteoforms in each fraction. N-terminally truncated proteoforms, where the hydrophilic region of the peptide is removed leaving the mid-domain and hydrophobic C-terminus, were more enriched in the insoluble fraction relative to the soluble aggregates^26^. In contrast, enriched species in the soluble Aβ aggregate fraction were proteoforms with nearly the entire hydrophobic C-terminus truncated. One proteoform, Aβ11–34, contained both N- and C-terminal truncations, which has not been described previously. Aβ1–34 was 5.6-fold more abundant in the soluble aggregates fraction than in the more insoluble material (Fig. 4, Supplementary Fig. 6). Aβ1–20 and Aβ11–34, both previously reported in the CSF^22, 27^, were 4- and 2.9-fold more abundant in the more soluble material, respectively (Supplementary Figs 7 and 8). Notably, Aβ1–42 and Aβ1–40 appeared to be the least significantly differentially expressed between the two fractions, remaining well within the ±2-fold change threshold (Fig. 4).

Next we sought to determine if the post-mortem interval (PMI) could affect Aβ proteoform truncations and PTMs (Supplementary Fig. 9). The signal intensity (*i*.*e*., relative abundance) of Aβ2–42 and Aβ3–42 showed a negative correlation in the soluble aggregates as a function of PMI and no significant effect in the more insoluble aggregates. This is the opposite of the increase that would be expected if these truncations were an artefact of PMI. The N-terminal truncations Aβ4–42, Aβ4–40, Aβ5–42 Amm., Aβ5–42, Aβ8–42, and Aβ8–40 all demonstrated no significant correlation with PMI in either the soluble or more insoluble aggregates (Supplementary Fig. 9N-U). The C-terminally truncated proteoforms found with high relative expression in the soluble aggregates – Aβ1–34, Aβ1–20, and Aβ11–34 – demonstrated no significant correlation with PMI (Supplementary Fig. 9A,G,V). Proteoform Aβ1–31 also showed no significant correlation. Intriguingly, the truncated proteoform Aβ11–42, in both the un-oxidized and oxidized state, and as well as Aβ p11–42 showed a high positive correlation in the more insoluble aggregates with PMI, but a high to moderate negative trend in the soluble aggregates (Supplementary Fig. 9Y,Z). Given that the trend for increased relative abundance of Aβ11–42 occurs irrespective of the oxidation status of methionine 35, we postulate that this may be due to the N-terminal truncation rather than oxidation. This may reflect residual BACE1 activity in the brain even after death^28^ for which neither our standard inhibitors (aprotinin and leupeptin) nor the protease cocktail, would block or inactivate (Supplementary Fig. 2). However, these results should be interpreted with caution due to the small sample size; only two participants had PMI of ≥12 hours.

To summarize, we have demonstrated that human brain soluble Aβ aggregates consist of an extraordinarily heterogeneous population of Aβ proteoforms. Other human brain proteins such as alpha-synuclein have not been reported to contain such substantial heterogeneity^29^, though tau post-translational modifications are protean in transgenic mouse brains^30^. Our results provide the first direct information regarding the proteoform constituents of soluble Aβ aggregates in human AD brain of which we are aware. Soluble Aβ aggregates may be important therapeutic targets, as they are believed to be the most toxic form of Aβ. Notably, human brain soluble Aβ aggregates have been reported to be substantially more toxic than similarly sized synthetic Aβ aggregates^31, 32^. These results suggest the hypothesis that previously unrecognized Aβ proteoforms may contribute to the modulation of aggregate toxicity in AD. However, these results should be interpreted with caution as some of these Aβ proteoforms have also been detected in cognitively intact individuals with Aβ and tau pathology^33^. The lack of control (*i*.*e*., unaffected) samples in this study limits the interpretation of these findings. The specificity of these Aβ proteoforms cannot be determined through comparison to similarly prepared non-AD brain or early stage AD samples. Clearly, a great deal of additional investigation with a larger cohort will be required to determine the clinical relevance, aggregation properties, and toxicity of the Aβ proteoforms described here. Cross-sectional study of Aβ proteoforms to map disease specificity (control *versus* AD), disease progression specificity (control *versus* mild *versus* severe AD) is the next logical step, as this will inform future efforts in animal models of the disease. The extent to which the most relevant Aβ proteoforms are recapitulated in animal models of AD-related pathology remains to be determined and may be of great importance for preclinical therapeutic development.

## Methods and Materials

### Regulatory Compliance

All protocols were carried out in accordance with the Charles F. and Joanne Knight Alzheimer’s Disease Research Center and Washington University guidelines. This specific study was approved by the Knight Alzheimer’s Disease Research Center tissue committee. All donors or their surrogates gave informed consent for their brains to be used for research studies.

### Human Tissue

Clinically and neuropathologically well-characterized human brain tissue samples were obtained from the Charles F. and Joanne Knight Alzheimer’s Disease Research Center (knight ADRC), Washington University School of Medicine, Saint Louis, Missouri. At the time of death, informed consent was obtained from the next of kin in accordance with the local Institutional Review Board. Cognitive status at expiration was determined using a validated retrospective post-mortem interview with an informant to establish the Clinical Dementia Rating (CDR)^34^. We used frozen tissue from the frontal lobe (Brodmann areas 8/9) of severely demented participants with Alzheimer’s disease dementia (CDR3, mean age at death = 84 ± 6.6 yrs; n = 6; mean post-mortem interval = 11.4 ± 4.8 hours (Table 1). In addition, parietal lobe was used for method development in addition to frontal lobe. Routinely, the right cerebral hemisphere was coronally sliced at 1 cm intervals and frozen by contact with pre-cooled Teflon®-coated aluminum plates and temperature equilibrated by immersion into liquid nitrogen vapor in a cryo-vessel. Following freezing, tissues were placed in Ziploc® storage bags and stored in freezer at −80 °C^35^.

### Amyloid-beta (Aβ) Extraction and Separation of Soluble Aggregates from Insoluble Material

Complete methods are described in Esparza *et al*.^20^. Briefly, 1–2 g of frozen CDR3 (severe AD) frontal cortical samples, including both gray and white matter, were weighed, stripped of pia mater, leptomeningeal, and intraparenchymal vessels to the fullest extent possible. and dounce homogenized at a 10:1 buffer volume:tissue weight ratio using a constant 25–40 manual strokes. Homogenization buffer consisted of ice-cold 1X phosphate buffered saline (PBS) (137 mM sodium chloride, 7.76 mM sodium phosphate dibasic, 2.17 mM monopotassium phosphate, 2.7 mM potassium chloride) with 0.45% (w/v) (3-((3-cholamidopropyl) dimethylammonio)−1-propanesulfonate) (CHAPS) and 1X protease inhibitor (2 ug/mL aprotinin and 1 ug/mL leupeptin). The resulting homogenate was rotated for 15 min at 4 °C before centrifugation at 17,000 × *g* for 30 min in a Sorvall RC 5B centrifuge with a SS-34 rotor. The supernatant was centrifuged in Beckman Optima XPN-100 centrifuge with a Ti70.1 rotor for 60 min at 100,000 × *g* at 4 °C, and the pellet (insoluble material; *see below*) was resolubilized in 5 M guanidine hydrochloride. The supernatant of the 100,000 x *g* spin was centrifuged for 60 min at 475,000 × *g* at 4 °C with a 70% sucrose cushion. The bottom layers (~2 mL) atop of the sucrose cushion (*see* Fig. 1a) were used for analysis of soluble Aβ aggregates. These methods have been demonstrated to preserve the size distribution of soluble Aβ aggregates present in PBS lysates, and do not result in artefactual aggregation of monomeric Aβ^20^. All pipet tips and tubes were blocked with 2% bovine serum albumin to significantly reduce non-specific loss of Aβ.

### Purification of Soluble Aβ Aggregates

The layers atop the 70% sucrose cushion of the 475,000 × *g* spin were immunoprecipitated with 100 uL/mL of a 50% slurry of beads conjugated to the monoclonal antibodies HJ3.4 and HJ5.1 overnight (22 hrs) at 4 °C. HJ3.4 binds the N-terminus of canonical Aβ, and HJ5.1 binds a mid-domain epitope. Beads were washed 15 times in (1 mL each) 1X PBS and eluted with formic acid at room temperature for 15 min. Total protein and Aβ concentrations were determined by NanoOrange (Molecular Probes, Eugene, OR) and ELISA, respectively. 5 ng of total Aβ from each participant sample was dried to completeness *in vacuo*. The 2D Clean-Up Kit (GE Healthcare, Piscataway, NJ) was used to desalt and delipidate the samples. Precipitated samples were subjected to C_8_ TopTips (Glygen, Columbia, MD) to separate full-length proteins from Aβ peptides (Supplementary Fig. 1, Supplementary Tables 1 and 2). C_8_ TopTips were conditioned in 60% ACN/ 0.05% TFA (w/v) and equilibrated in 0.05% TFA (*aq*., v/v) three times each for 1 min at 2,000 rotations-per-minute (rpm). Next, precipitated samples were resuspended in 80 uL of neat formic acid and applied to the C_8_ TopTips and spun for 1 min at 2,000 rpm. The flowthrough was collected and dried to completeness *in vacuo* and stored at −80 °C until analysis by nLC-MS/MS.

### Purification of Insoluble Aβ Aggregates

The pellet of the 100,000 × *g* spin was resolubilized in 5 M guanidine hydrochloride (pH 8.0) overnight at 4 °C. The resulting guanidine solubilized 100 k pellet was centrifuged in a MicroCL 17 R centrifuge at 17,000 × *g* to remove any guanidine insoluble material. Next, the supernatant was diluted 1:10 (0.5 M guanidine, final concentration) in 1X PBS and immunoprecipitated with 100 uL/mL of a 50% slurry of immobilized monoclonal antibodies HJ3.4 and HJ5.1 overnight (22 hrs) at 4 °C. Beads were washed 15 times in 1X PBS (1 mL each) and eluted 3X with 100 uL formic acid at room temperature for 5 min each. Total protein and Aβ concentrations were determined by NanoOrange (Molecular Probes, Eugene, OR**)** and ELISA, respectively^20^. 5 ng of total Aβ from each patient sample was dried to completeness *in vacuo*. The 2D Clean-Up Kit (GE Healthcare, Piscataway, NJ) was used to desalt and delipidate the samples. Precipitated samples were subjected to C_8_ TopTips (Glygen, Columbia, MD) to separate full-length proteins from Aβ peptides (Supplementary Fig. 1, Supplementary Tables 1 and 2). C_8_ TopTips were condition in 60% ACN/0.05% TFA (v/v) and equilibrated in 0.05% TFA (*aq*., v/v) three times each for 1 min at 2,000 rpm. Next, precipitated samples were resuspended in 80 uL of neat formic acid and applied to the C_8_ TopTip and spun for 1 min at 2,000 rpm. The flowthrough was collected and dried to completeness *in vacuo* and stored at −80 °C until analysis by nLC-MS/MS.

### Aβ ELISA

The Aβ1-x ELISA was performed as previously described^20^. Briefly, mouse monoclonal HJ5.1 (mid-domain) was used to coat 96-well Nunc MaxiSorp plates (464718, Nalge Nunc, Rochester, NY) at 20 μg/mL in a carbonate buffer (35 mM sodium bicarbonate, 16 mM sodium carbonate, 3 mM sodium azide, pH 9.6) using 100 μl/well overnight at 4 °C. After washing 5x and blocking in 2% bovine serum albumin (BSA, A7030, Sigma-Aldrich, St. Louis, MO) in 1X PBS for 30 min at room temperature, samples and standards (Aβ1–40 on a 8-point standard curve) were plated in dilution buffer^20^ and incubated overnight at 4 °C. Samples and standards were developed by incubating in sequence with biotinylated HJ3.4 (canonical N-terminus) at 100 ng/mL for 1 hour at room temperature, streptavidin HRP-20 (65R-S103PHRP, Fitzgerald, Acton, MA) for 30 min at room temperature, and finally 3, 3′, 5, 5′ – tetramethylbenzidine (TME) (T5569, Sigma-Aldrich, St. Louis, MO) for measurement on a BioTek Synergy 2 plate reader at 650 nm as previously described^20^.

### Protease Inhibitor Control

While the truncation seen is not likely a post-mortem artifact, we used only two protease inhibitors in our homogenization buffer instead of a cocktail of inhibitors, which would protect against a broader spectrum of protease activities. To evaluate the possible impact of using only two protease inhibitors in the truncation profile of Aβ, we analyzed a representative sample (Pt1) using our standard inhibitors (aprotinin and leupeptin) or Halt Protease and Phosphatase Inhibitor Cocktail (78443, Thermo Fisher Scientific) at 1X (final concentration). Three grams of frontal tissue was diced (on ice) and split evenly for homogenization in either the standard buffer or the cocktail buffer and processed for insoluble and soluble fractions as described above (Supplementary Fig. 2). Mass spectrometry analysis we performed as described below. Spider search results with a score (−10logP) of 31.7 or higher (an estimated FDR value of 2.3% at the peptide level). Only truncated proteoforms were considered for further analysis.

### nLC-MS/MS

Samples from each individual CDR3 participant (n = 6, biological replicates) across the HMW soluble and insoluble Aβ fractions were split into two technical replicates (of 5 ng total Aβ as measured by ELISA) with the exception of Participant 4 (Pt4), which did not have enough material for a second technical replicate. All samples were prepared (precipitation and C_8_ SPE) in two block-randomized sets (https://www.random.org)^36^ for each set of participant replicates across two days. Samples were resuspended in 1%/10%/5% FA/ACN/MeOH (v/v) and analyzed by non-stop nLC-MS/MS in a block-randomized fashion^36^ – to eliminate systemic bias due to run order – for a total of 22 Thermo.raw files on a LTQ-Orbitrap Fusion (Thermo Fisher Scientific). Throughout data acquisition, quality assurance/quality control (QA/QC) standard of 20 fmoles BSA (#P8108S, New England BioLabs, Ipswich, MA) was injected every 12–18 hrs to monitor instrument drift and variability over time. 29 BSA peptides from each interwoven QA/QC run were analyzed with AutoQC for instrument performance metrics^37^ (Supplementary Fig. 3).

Separations were performed using an online NanoAcquity UPLC (Waters). The chromatographic separation was performed on an ACQUITY UPLC HSS T3 (360 μm OD × 75 μm ID) column packed with 10 cm C_18_ (1.8 μm, 100 Å, Waters) at 300 nL/min and heated to 60 °C. Mobile phases were 0.1% FA in water (A) and 0.1% FA in ACN (B). Samples were eluted from the column with the gradient ramped to 35% B over 65 min and further increased to 95% B over 8 min and held for an additional 6 min. Total run time, including column equilibration, sample loading, and analysis was 89 min. The mass spectrometer was operated in data-dependent mode to automatically switch between MS and MS/MS acquisition. The survey scans at mass-charge ratio (*m*/*z*) 400–2000 (MS) were acquired in the Orbitrap at high resolution (60,000 at *m*/*z* 400) in profile mode, and the MS/MS spectra were acquired in the Orbitrap (15,000 at *m/z* 400) in centroid mode using XCalibur, version 3.0 (Thermo Fisher Scientific). Ion injection times for the MS and MS/MS scans were 500 ms each. The automatic gain control targets were set as 2 × 10^5^ for MS and MS/MS in the Orbitrap. The most abundant precursor ions from each MS scan were sequentially isolated and fragmented in the Orbitrap using HCD (isolation width 2.0 Da, normalized collision energy 30%, activation *Q* 0.250, and activation time 10 ms) within a 3 sec duty cycle (TopSpeed method). Dynamic exclusion (±10 ppm relative to precursor ion *m/z*) was enabled with a repeat count of one, a maximal exclusion list size of 500, and an exclusion duration of 60 s. Monoisotopic precursor selection (MIPS) was enabled and unassigned ions were rejected.

### Mass Spectrometry Data Processing

MS files (.raw) were imported into PEAKS (version 8, Bioinformatics Solutions Inc., Waterloo, ON) and searched against a UniprotKB/SwissProt Human database of reviewed, canonical sequences (October 2015; 20,204 entries) appended with the cRAP contaminant database (January 2015 version, The Global Proteome Machine, www.thegpm.org/cRAP/index.html). Precursor ion mass tolerance was set to 10 ppm, and fragment mass tolerance was 0.1 Da with no enzyme specificity. All modifications in the UniMod database (http://www.unimod.org) were considered in the PEAKS search. PEAKS automatically generates a decoy-fusion database, which appends a decoy sequence to each protein identification for the calculation of FDR^38^. The Spider search results with a score (−10logP) of 31.9 or higher (an estimated FDR value of 2.9% at the peptide level) for the CDR3 cohort data. Spider is an algorithm tool within PEAKS, which we utilized to search peptide spectrum matches not identified by the database search by altering the amino acids systematically at each residue until a new, better peptide sequence is constructed from the MS/MS data^39^. All non-Aβ peptides and those Aβ proteoforms containing formylation were removed. Immunoprecipitation elution was in neat formic acid (Sigma #94318); thus, formylation occurring endogenously or by the elution conditions is indistinguishable. Further, samples were resuspended in MS buffer containing 5% methanol (v/v) to maintain solubility of Aβ, which in combination with 1% FA could lead to artefactual methylation via Fischer esterification.

In the CDR3 cohort, 27 Aβ proteoforms were identified meeting the criteria described above. However, we sought reproducible identification of the 27 Aβ proteoforms on an independent platform. The filtered list of Aβ proteoforms were *i*) assigned identification numbers by a third party who had no part in the initial analysis and *ii*) transformed into chemical formulas. This chemical formula list was queried against each.raw file with National Resource for Top-Down Proteomics (NRTDP) pipeline version 1.3 at ± 10 ppm precursor ion mass tolerance and a 4 min retention time window alignment (Supplementary Fig. 4). If a given proteoform was not identified by both platforms (PEAKS and NRTDP), it was removed from analysis; 26 out of 27 were reproducibly identified on both platforms. MS intensity was calculated across all peaks within an isotopic cluster extracted with Skyline for each proteoform identified in Fig. 2 and exported as an Excel file.

### Differential Mass Spectrometry (dMS)

In this method relative quantitation is performed from the full-scan MS. The high-resolution MS provides *m/z*, retention time, charge state, and relative abundance (intensity) of precursor ion across multiple samples for comparative analysis^40–43^. A table of proteoform intensities was imported into a custom SAS script for analysis. To detect differential (label-free) abundance from the intensities data^40^, the MS intensity measures (calculated as described above) for each proteoform were standardized to Z-scores across all measures of that proteoform. Different charge states were treated as independent measures of the same proteoform as reflected in the increased degrees of freedom for certain tests (Supplementary Table 3).

ANOVA based on a hierarchical linear model (HLM) with replicates nested within patients, and patients treated as random effects, was used to test the fixed effect difference between soluble aggregate Aβ and more insoluble Aβ fractions. All calculations were done using SAS PROC MIXED with restricted maximum likelihood estimations (SAS Institute, Cary, NC) and type 3 sums of squares (where appropriate). The HLM was used to test for differences in mean intensity between CDR3 soluble and insoluble Aβ fractions, while allowing each biological replicate to have its own overall mean. Each *p*-value of the resulting 26 F tests was corrected for multiple testing (*q*-value), and those with an FDR of ≤0.05 were considered significant^44^. Next, the same model was run on the log_2_-converted raw intensities. The difference in estimated mean between aggregate Aβ and more insoluble Aβ fractions in these tests was taken as an estimate of the overall fold change within the fractions. Thus two separate ANOVA analyses are run, the first to test the statistical significance of abundances of proteoforms between fractions, and the second to estimate effect size^25, 45^. The mass spectrometric data have been deposited in ProteomeXchange (http://proteomecentral.proteomexchange.org) via the PRIDE partner repository^46^ with the data set identifier PXD005119.

### Data Visualization

Analyses were performed and visualized with Excel 2013, PRISM (version 7) for correlation analysis of signal intensity and PMI and graphing volcano plots or SAS (version 9.4) for hierarchical linear modeling.

## Electronic supplementary material

Supplementary Information

Supplementary Table 3
