# Supplementary material for: Diversity of Amyloid-beta Proteoforms in the Alzheimer’s Disease Brain
Source: Sci Rep. 2017 Aug 25;7:9520. doi: 10.1038/s41598-017-10422-x (PMC5572664; doi:10.1038/s41598-017-10422-x)
Supplement: Supplementary file 1 — Supplementary Information [file 41598_2017_10422_MOESM1_ESM.pdf]

Diversity of Amyloid-beta Proteoforms in the Alzheimer's Disease Brain

Norelle C. Wildburger, Thomas J. Esparza, Richard D. LeDuc, Ryan T. Fellers, Paul M. Thomas, Nigel J. Cairns, Neil L. Kelleher, Randall J. Bateman, David L. Brody

SUPPLEMENTARY INFORMATION:

SUPPLEMENTARY FIGURES

Supplementary Fig. 1: Separation of Aβ from other co-immunoprecipitated proteins

Supplementary Fig. 2: Protease inhibitor comparison

Supplementary Fig. 3: System performance over time during mass spectrometry data acquisition

Supplementary Fig. 4: Schematic of data analysis

Supplementary Fig. 5: Additional mass spectrometry data for Aβp11-42 and Aβ4-42 detected in human AD brain

Supplementary Fig. 6: Tandem mass spectrometry data for proteoform Aβ1-34

Supplementary Fig. 7: Tandem mass spectrometry data for proteoform Aβ1-20

Supplementary Fig. 8: Tandem mass spectrometry data for proteoform Aβ11-34

Supplementary Fig. 9: Soluble aggregate Aβ and more insoluble Aβ proteoform correlations with post-mortem interval

SUPPLEMENTARY TABLES

Supplementary Table 1: Representative biochemical purifications of soluble high molecular weight Aβ aggregates by immunoprecipitation

Supplementary Table 2: Aβ C_8 solid phase extraction separation characteristics

SUPPLEMENTARY DISCUSSION

Summary

Relationship to previous studies

Advantages of our approach

Limitations

Priorities for future research

Implications

SUPPLEMENTARY REFERENCES

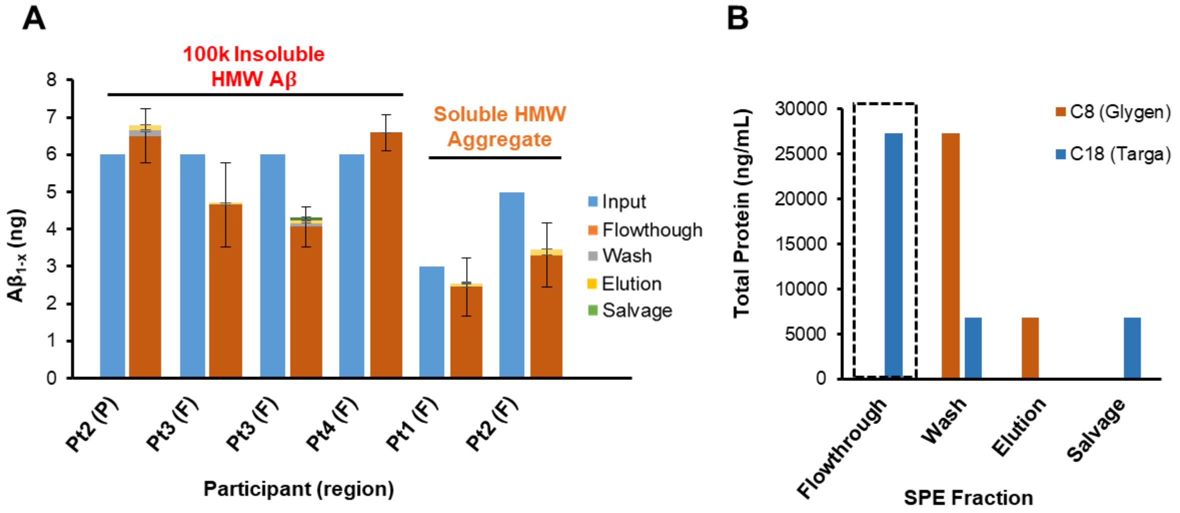

Supplementary Figure 1. Separation of Aβ from other co-immunoprecipitated proteins. A. Quantitative tracking of total Aβ during each step of the C_8 extraction process. Quantitative tracking of total Aβ monomer equivalents during each step of the C_8 SPE process. Input, starting amount of Aβ placed in the C_8 SPE tip after conditioning and equilibration with 60% ACN/0.05% TFA and 0.05% TFA (*aq*), respectively; Flowthrough, unbound input material eluting through the tip; Wash, material eluting after wash steps in 10% ACN/10% TFA; Elution, material eluting after wash with 60% ACN/10% TFA; Salvage, material remaining in the sample tube salvaged with neat formic acid. Pt., participant; P, parietal lobe; F, frontal lobe. **B**. Representative quantitative tracking of total protein during each step of the C_8 extraction process. Total protein levels were undetectable (below 156.2 ng/mL, by NanoOrange) in the flowthrough where Aβ elutes (panel A). As comparison, Targa® C_{18} SPE micro-spin columns demonstrate release of nearly all protein in the flowthrough, where Aβ also elutes.

A

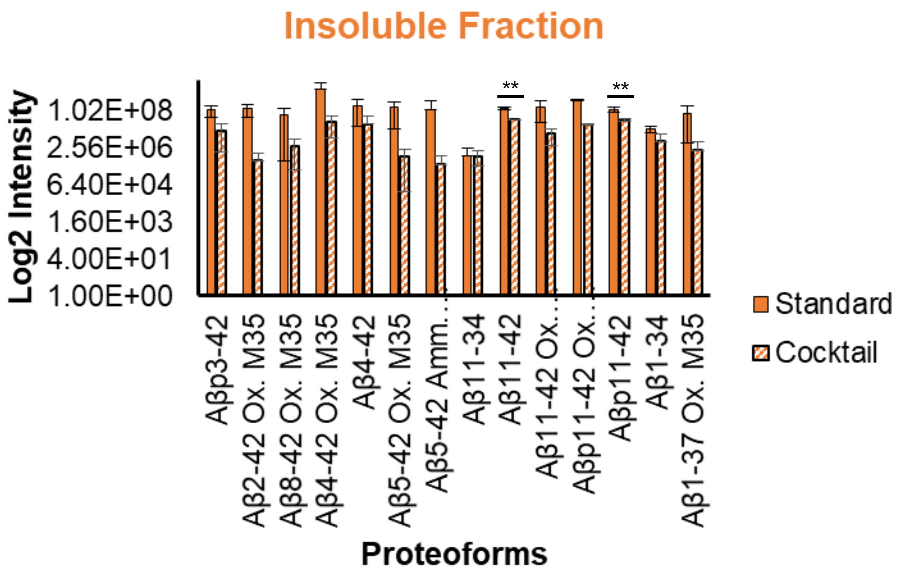

B

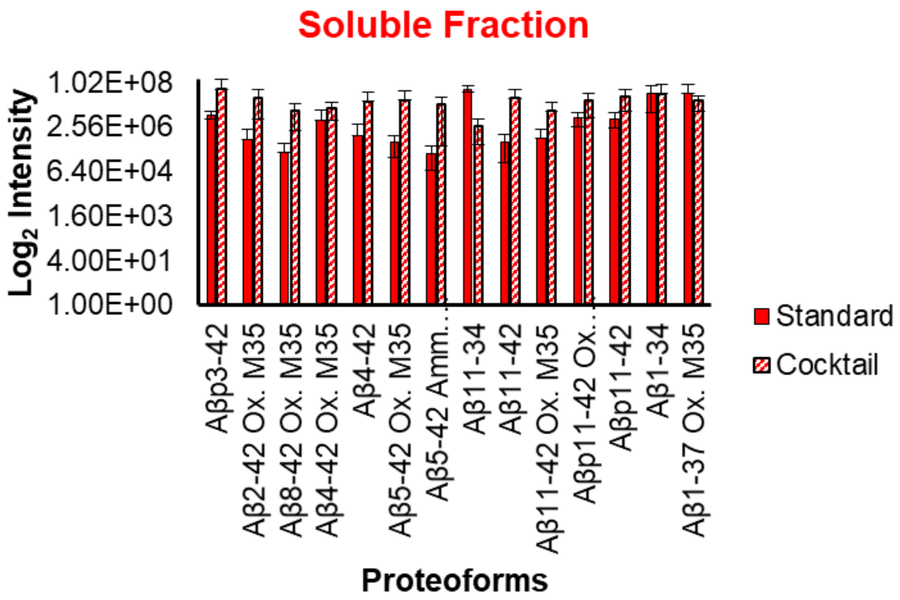

Supplementary Figure 2. Protease inhibitor comparison. A. Logarithmic MS1 intensity of truncated Aβ proteoforms in the more insoluble fraction (100K) from a representative participant sample. **B.** Logarithmic MS1 intensity of truncated Aβ proteoforms in the soluble fraction (475K) from a representative participant sample. Standard buffer conditions with two protease inhibitors (aprotinin and leupeptin, see *Methods and Materials*) *versus* homogenization buffer with 1X protease and phosphatase inhibitor cocktail. Data is represented as mean ± S.D. of duplicated injections. ** $q < 0.01$ (q-values (q is a false discovery rate corrected p-value, where $q \leq 0.05$ is considered statistically significant).

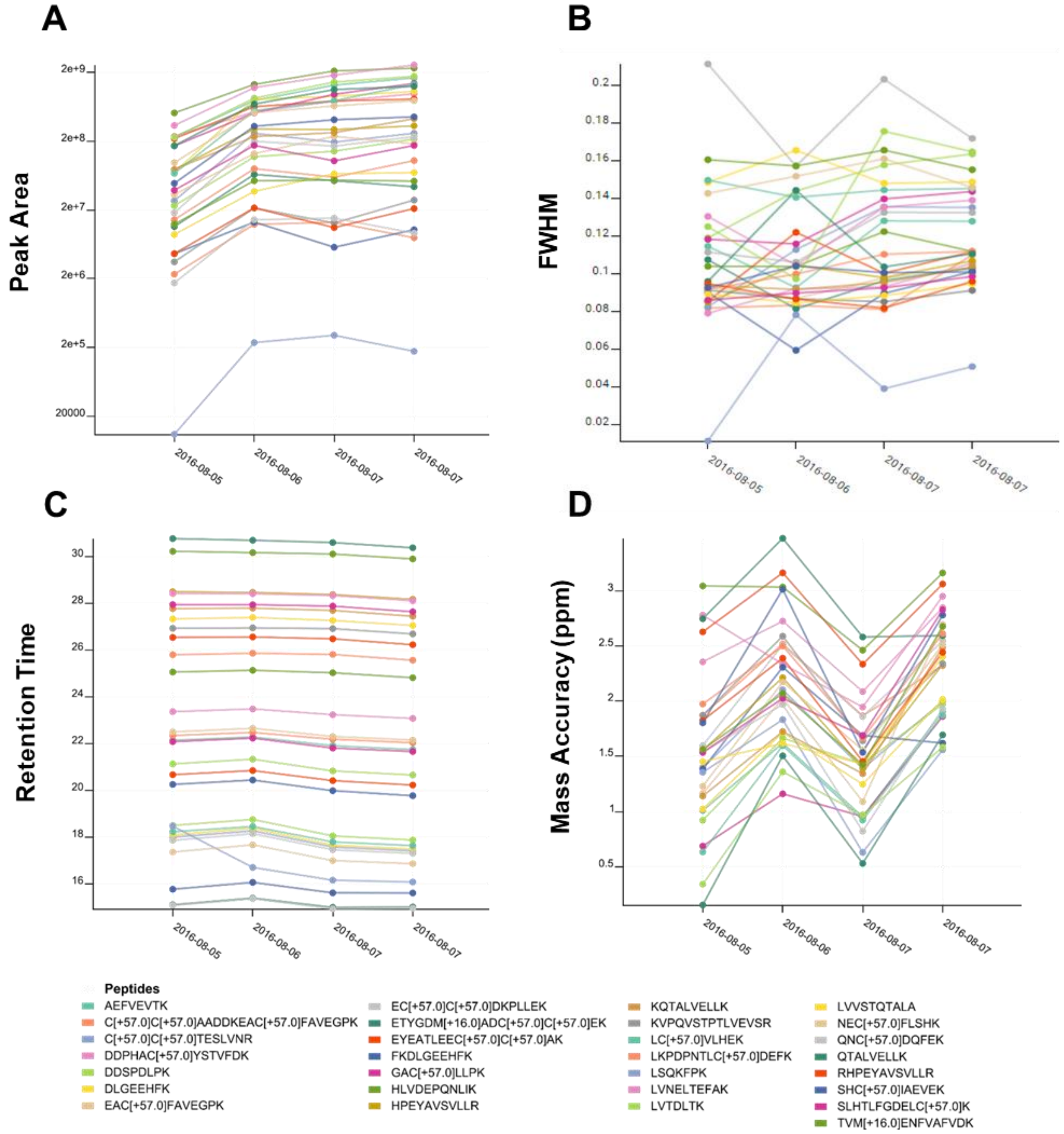

Supplementary Figure 3. System performance over time during mass spectrometry data acquisition. A. Peak area measurements (from full scan MS; log scale) of 29 bovine serum albumin (BSA) peptides injected throughout the CDR3 cohort data acquisition at 20 fmole for quality assurance/quality control (QA/QC) metrics over the four-day course of the CDR3 cohort data-dependent acquisition (DDA) experiment. **B.** Peak full width half maximum (FWHM) measurements of 29 BSA peptides. **C.** Retention time (minutes) metric of 29 BSA peptides. Retention time drifts were no more than ±1 min with the exception of peptide LSQKFPK (from 18.5 min to 16.1-16.7 min) in the first run, likely due to column equilibration at the beginning. **D.** Mass accuracy in parts-per-million (ppm) of 29 BSA peptides over the four-day course of the CDR3 cohort DDA experiment. Mass accuracy did not demonstrate a substantial drift *(e.g.,* ±15 ppm). These system suitability metrics for DDA (*i.e.*, untargeted) proteomic experiments are extracted from the data directly and do not require database searches (*i.e.,* identification-free metrics). These metrics allow determination of sub-optimal instrument performance which, in a untargeted proteomics experiments, can waste biological samples and lead to incorrect conclusions. Overall these metrics are stable throughout the experiment.

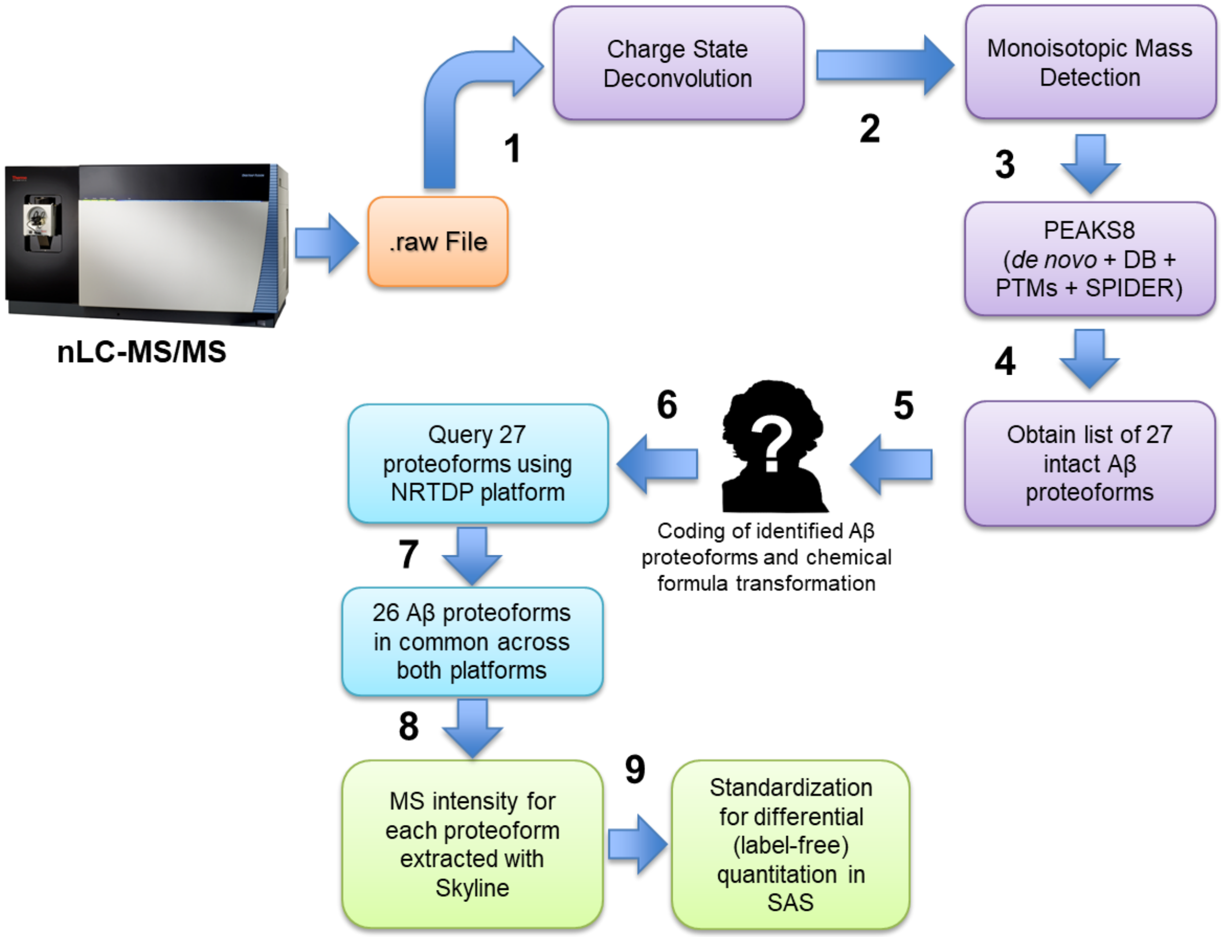

Supplementary Figure 4. Schematic of data analysis. After top-down nLC-MS/MS data acquisition, Thermo .raw files were imported into PEAKS8 where the spectra underwent (**1**) charge state deconvolution and (**2**) mass detection. (**3**) Refined *m/z* data was searched in PEAKS8 successively by *de novo* sequencing, human canonical proteome database (DB), PEAKS PTM using all PTMs contained in the UniMod database, and finally SPIDER to match unidentified spectra by altering the amino acids systematically at each residue until a new, better peptide sequence was constructed from the MS/MS data. This resulted in 27 identified Aβ proteoform (**4**), which were coded by a 3rd party (**5**) and searched independently in the NRTDP platform (**6**). One Aβ proteoforms identified in PEAKS failed to be recapitulated with NRTDP (**7**). Mass spectrometry data for each of the 26 proteoforms was extracted with Skyline across all charge states for each proteoform (**8**). Data was standardized and analyzed in SAS (**9**).

A

#	b	b-H2O	b-NH3	a	b (2+)	a (2+)	Seq	y	y-H2O	y-NH3	y (2+)	#
1	112.04	94.03	95.01	84.04	56.52	42.52	E(-18.01)					32
2	211.15	193.10	194.08	183.11	106.05	92.06	V	3205.76	3187.74	3188.73	1603.38	31
3	348.17	330.16	331.14	320.17	174.58	160.59	H	3106.69	3088.68	3089.66	1553.84	30
4	485.23	467.22	468.20	457.23	243.11	229.12	H	2969.63	2951.62	2952.60	1485.31	29
5	613.28	595.27	596.33	585.29	307.14	293.14	Q	2832.57	2814.56	2815.54	1416.78	28
6	741.40	723.37	724.35	713.41	371.19	357.19	K	2704.51	2686.50	2687.48	1352.76	27
7	854.46	836.45	837.44	826.47	427.73	413.73	L	2576.42	2558.41	2559.39	1288.71	26
8	953.53	935.52	936.51	925.54	477.27	463.27	V	2463.33	2445.32	2446.30	1232.13	25
9	1100.60	1082.59	1083.57	1072.61	550.80	536.81	F	2364.26	2346.18	2347.18	1182.63	24
10	1247.67	1229.66	1230.64	1219.67	624.33	610.24	F	2217.19	2199.18	2200.17	1109.10	23
11	1318.70	1300.70	1301.68	1290.71	659.85	645.86	A	2070.13	2052.12	2053.10	1035.56	22
12	1447.74	1429.74	1430.72	1419.78	724.37	710.38	E	1999.09	1981.08	1982.06	1000.04	21
13	1562.77	1544.77	1545.75	1534.78	781.89	767.89	D	1870.05	1852.04	1853.02	935.52	20
14	1661.84	1643.83	1644.82	1633.85	831.47	817.42	V	1755.02	1737.01	1737.99	878.01	19
15	1718.87	1700.85	1701.84	1690.87	859.93	845.94	G	1655.95	1637.94	1638.92	828.48	18
16	1805.91	1787.89	1788.87	1777.90	903.45	889.45	S	1598.93	1580.92	1581.90	799.96	17
17	1919.94	1901.93	1902.91	1891.95	960.47	946.47	N	1511.90	1493.89	1494.87	756.45	16
18	2048.03	2030.02	2031.01	2020.04	1024.52	1010.61	K	1397.85	1379.84	1380.83	699.43	15
19	2105.08	2087.05	2088.03	2077.06	1053.03	1039.03	G	1269.76	1251.75	1252.73	635.38	14
20	2176.10	2158.08	2159.07	2148.10	1088.55	1074.55	A	1212.74	1194.73	1195.71	606.87	13
21	2289.18	2271.17	2272.15	2261.18	1145.11	1131.10	I	1141.62	1123.69	1124.67	571.31	12
22	2402.26	2384.25	2385.23	2374.27	1201.65	1187.63	I	1028.62	1010.61	1011.59	514.81	11
23	2459.28	2441.25	2442.26	2431.28	1230.14	1216.14	G	915.53	897.51	898.51	458.24	10
24	2572.36	2554.34	2555.35	2544.38	1286.68	1272.69	L	858.51	840.50	841.48	429.76	9
25	2703.41	2685.40	2686.38	2675.41	1352.23	1338.21	M	745.38	727.41	728.40	373.21	8
26	2802.48	2784.47	2785.45	2774.48	1401.74	1387.74	V	614.30	596.33	597.36	307.69	7
27	2859.50	2841.49	2842.47	2831.50	1430.25	1416.25	G	515.26	497.31	498.29	258.16	6
28	2916.52	2898.51	2899.49	2888.52	1458.78	1444.76	G	458.24	440.29	441.27	229.65	5
29	3015.59	2997.58	2998.56	2987.59	1508.29	1494.30	V	401.22	383.27	384.26	201.09	4
30	3114.66	3096.65	3097.63	3086.66	1557.83	1543.83	V	302.15	284.20	285.18	151.60	3
31	3227.74	3209.73	3210.71	3199.75	1614.37	1600.37	I	203.14	185.17	186.12	102.07	2
32							A	90.05	72.04	73.03	45.53	1

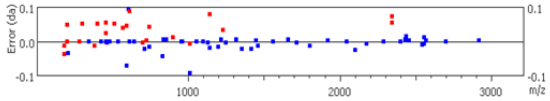

B

#	b	b-H2O	b-NH3	a	b (2+)	a (2+)	Seq	y	y-H2O	y-NH3	y (2+)	#
1	148.0762	130.0657	131.0492	120.0813	74.5381	60.5406	F					39
2	304.1773	286.1969	287.1507	276.1823	152.5887	138.5912	R	4050.1018	4032.0913	4033.0747	2025.5509	38
3	441.2327	423.2257	424.2093	413.2412	221.1181	207.1206	H	3894.0005	3875.9900	3876.9734	1947.5002	37
4	556.2634	538.2526	539.2362	528.2682	278.6316	264.6341	D	3756.9417	3738.8638	3739.9146	1878.9708	36
5	643.2952	625.2847	626.2682	615.2811	322.1476	308.1501	S	3641.9148	3623.9043	3624.8877	1821.4574	35
6	700.3167	682.3061	683.2897	672.3217	350.6583	336.6608	G	3554.8828	3536.8723	3537.8557	1777.9414	34
7	863.3800	845.3694	846.3530	835.3850	432.1900	418.1925	Y	3497.8613	3479.8508	3480.8342	1749.4307	33
8	992.4162	974.4120	975.3956	964.4276	496.7160	482.7138	E	3334.7979	3316.7874	3317.7708	1667.8989	32
9	1091.4861	1073.4805	1074.4640	1063.4960	546.1598	532.2480	V	3205.7554	3187.7449	3188.7283	1603.3777	31
10	1228.5648	1210.5394	1211.5229	1200.5549	614.7750	600.7786	H	3106.6870	3088.6765	3089.6599	1553.8435	30
11	1365.6030	1347.5983	1348.5818	1337.6138	683.3044	669.3197	H	2969.6279	2951.6174	2952.6008	1485.3140	29
12	1493.6959	1475.6569	1476.6404	1465.7002	747.3337	733.3362	Q	2832.5691	2814.5586	2815.5420	1416.7845	28
13	1621.7662	1603.7518	1604.7354	1593.7673	811.3812	797.3837	K	2704.5105	2686.5000	2687.4834	1352.7552	27
14	1734.8367	1716.8397	1717.8195	1706.8514	867.9232	853.9257	L	2576.4155	2558.4050	2559.3884	1288.7078	26
15	1833.9199	1815.9043	1816.8878	1805.9152	917.4574	903.4599	V	2463.3315	2445.3210	2446.3044	1232.1658	25
16	1980.9867	1962.9728	1963.9563	1952.9883	990.9916	976.9941	F	2364.2632	2346.2527	2347.2361	1182.6316	24
17	2128.0474	2110.0413	2111.0247	2100.0569	1064.5259	1050.5284	F	2217.1946	2199.1841	2200.1675	1109.0973	23
18	2199.0833	2181.0784	2182.0618	2171.0940	1100.0444	1086.0470	A	2070.1262	2052.1157	2053.0991	1035.5631	22
19	2328.1326	2310.1208	2311.1042	2300.1365	1164.5806	1150.5682	E	1999.0892	1980.9867	1982.0623	1000.0446	21
20	2443.1655	2425.1477	2426.1311	2415.1633	1222.0791	1208.0817	D	1870.0466	1852.0361	1853.0197	935.5233	20
21	2542.2290	2524.2161	2525.1995	2514.2317	1271.6133	1257.6396	V	1755.0197	1737.0092	1737.9927	878.0098	19
22	2599.2527	2581.2375	2582.2209	2571.2532	1300.1182	1286.1266	G	1655.9513	1637.9408	1638.9243	828.4756	18
23	2686.2573	2668.2698	2669.2532	2658.2854	1343.6595	1329.6427	S	1598.9298	1580.9193	1581.9028	799.9649	17
24	2800.3191	2782.3125	2783.2959	2772.3281	1400.6935	1386.6641	N	1511.8977	1493.8872	1494.8707	756.4489	16
25	2928.4180	2910.4075	2911.3909	2900.4231	1464.7090	1450.7115	K	1397.8549	1379.8444	1380.8279	699.4255	15
26	2985.4441	2967.4290	2968.4124	2957.4446	1493.2197	1479.2223	G	1269.7599	1251.7494	1252.7329	635.3799	14
27	3056.4766	3038.4661	3039.4495	3028.4817	1528.7383	1514.7408	A	1212.7384	1194.7279	1195.7114	606.8692	13
28	3169.5664	3151.5503	3152.5337	3141.5894	1585.2804	1571.2830	I	1141.7013	1123.6908	1124.6743	571.3198	12
29	3282.6421	3264.6343	3265.6177	3254.6499	1641.8224	1627.8250	I	1028.6173	1010.6067	1011.5903	514.8087	11
30	3339.6628	3321.6499	3322.6392	3311.6714	1670.3331	1656.3357	G	915.5332	897.5197	898.5062	458.2459	10
31	3452.7429	3434.7397	3435.7231	3424.7542	1726.8751	1712.8777	L	858.5117	840.5012	841.4847	429.7559	9
32	3583.7881	3565.7803	3566.7725	3555.7959	1792.3954	1778.3979	M	745.4277	727.4149	728.4007	373.2286	8
33	3682.8591	3664.8486	3665.8320	3654.8643	1841.9296	1827.9321	V	614.3348	596.3766	597.3729	307.6936	7
34	3739.8806	3721.8701	3722.8535	3711.8857	1870.4403	1856.4429	G	515.2547	497.3082	498.2917	258.1594	6
35	3796.9021	3778.8916	3779.8750	3768.9072	1898.9510	1884.9536	G	458.2459	440.2867	441.2327	229.6486	5
36	3895.9705	3877.9600	3878.9434	3867.9756	1948.4852	1934.4878	V	401.2197	383.2653	384.2649	201.1379	4
37	3995.0388	3977.0283	3978.0117	3967.0439	1998.0194	1984.0220	V	302.1538	284.1974	285.1804	151.6037	3
38	4108.1230	4090.1125	4091.0959	4080.1282	2054.5615	2040.5641	I	203.1398	185.1658	186.1248	102.0695	2
39							A	90.0549	72.0444	73.0279	45.5275	1

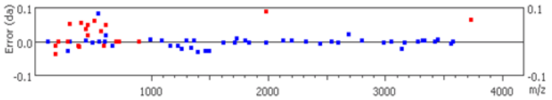

Supplementary Figure 5. Additional mass spectrometry data for Aβp11-42 and Aβ4-42 detected in human AD brain. **A**. The *b*-ion and *y*-ion table for Aβp11-42. Blue text indicates detected *b*-ions, red text indicated detected *y*-ions. Bottom graph indicates mass error relative to expected mass as a function of expected mass. **B.** The *b*-ion and *y*-ion table for Aβ4-42. Bottom graph indicates mass error relative to expected mass as a function of expected mass.

Supplementary Fig. 6A

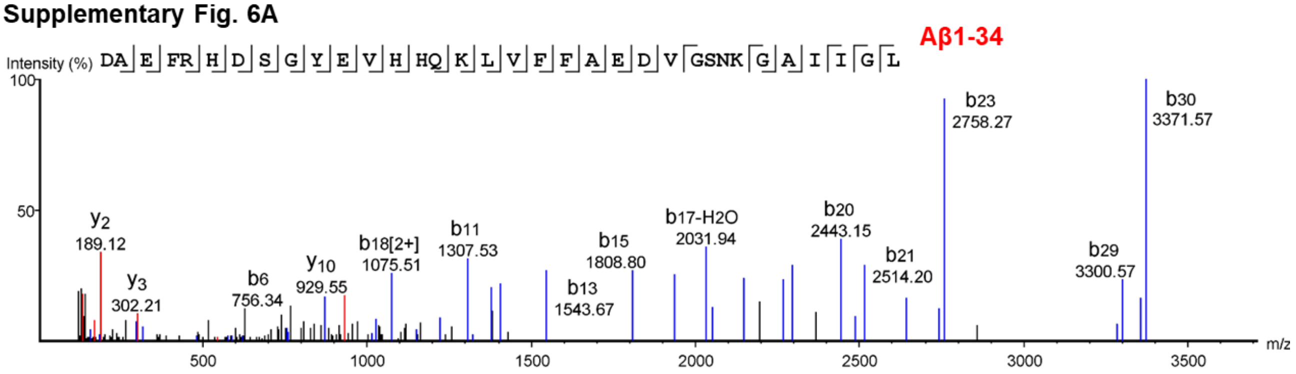

Supp. Fig. 6B

#	b	b-H2O	b-NH3	a	b (2+)	a (2+)	Seq	y	y-H2O	y-NH3	y (2+)	#
1	116.03	98.02	99.01	88.04	58.52	44.52	D					34
2	187.11	169.10	169.98	159.08	94.04	80.04	A	3670.84	3652.83	3653.81	1835.92	33
3	316.11	298.10	299.06	288.12	158.56	144.56	E	3599.80	3581.79	3582.78	1800.40	32
4	463.18	445.17	446.16	435.19	232.09	218.09	F	3470.76	3452.75	3453.73	1735.88	31
5	619.29	601.27	602.26	591.29	310.14	296.14	R	3323.69	3305.68	3306.66	1662.35	30
6	756.34	738.33	739.32	728.35	378.67	364.67	H	3167.59	3149.58	3150.56	1584.30	29
7	871.37	853.36	854.34	843.37	436.18	422.19	D	3030.53	3012.52	3013.50	1515.77	28
8	958.40	940.39	941.37	930.41	479.70	465.70	S	2915.50	2897.49	2898.48	1458.25	27
9	1015.42	997.41	998.40	987.43	508.21	494.21	G	2828.47	2810.46	2811.45	1414.74	26
10	1178.49	1160.48	1161.46	1150.49	589.75	575.75	Y	2771.45	2753.44	2754.42	1386.23	25
11	1307.53	1289.52	1290.50	1279.53	654.26	640.27	E	2608.39	2590.38	2591.36	1304.69	24
12	1406.59	1388.59	1389.57	1378.60	703.80	689.80	V	2479.35	2461.33	2462.32	1240.17	23
13	1543.67	1525.65	1526.63	1515.66	772.33	758.33	H	2380.28	2362.27	2363.25	1190.64	22
14	1680.72	1662.70	1663.69	1652.72	840.86	826.86	H	2243.22	2225.21	2226.19	1122.11	21
15	1808.80	1790.76	1791.75	1780.78	904.89	890.89	Q	2106.16	2088.15	2089.13	1053.58	20
16	1936.88	1918.86	1919.84	1908.87	968.93	954.94	K	1978.10	1960.09	1961.07	989.55	19
17	2049.96	2031.94	2032.93	2021.96	1025.50	1011.48	L	1850.01	1832.00	1832.98	925.50	18
18	2149.02	2131.01	2131.99	2121.03	1075.02	1061.01	V	1736.92	1718.91	1719.89	868.96	17
19	2296.12	2278.08	2279.06	2268.10	1148.56	1134.55	F	1637.85	1619.84	1620.83	819.43	16
20	2443.15	2425.15	2426.13	2415.16	1222.08	1208.08	F	1490.78	1472.77	1473.76	745.89	15
21	2514.20	2496.18	2497.17	2486.20	1257.60	1243.60	A	1343.72	1325.71	1326.69	672.36	14
22	2643.26	2625.23	2626.21	2615.24	1322.10	1308.12	E	1272.68	1254.67	1255.65	636.84	13
23	2758.27	2740.23	2741.24	2730.27	1379.63	1365.64	D	1143.64	1125.63	1126.61	572.32	12
24	2857.33	2839.32	2840.31	2829.34	1429.17	1415.17	V	1028.61	1010.60	1011.58	514.80	11
25	2914.35	2896.34	2897.33	2886.36	1457.68	1443.68	G	929.55	911.53	912.51	465.27	10
26	3001.39	2983.38	2984.36	2973.39	1501.19	1487.20	S	872.52	854.51	855.49	436.76	9
27	3115.43	3097.42	3098.40	3087.43	1558.21	1544.22	N	785.49	767.48	768.46	393.24	8
28	3243.52	3225.51	3226.50	3215.53	1622.26	1608.26	K	671.45	653.43	654.42	336.22	7
29	3300.57	3282.52	3283.52	3272.55	1650.77	1636.78	G	543.28	525.34	526.32	272.18	6
30	3371.57	3353.57	3354.59	3343.59	1686.29	1672.29	A	486.33	468.32	469.30	243.66	5
31	3484.67	3466.66	3467.64	3456.67	1742.83	1728.84	I	415.29	397.28	398.26	208.15	4
32	3597.75	3579.74	3580.72	3569.76	1799.38	1785.38	I	302.21	284.20	285.18	151.60	3
33	3654.77	3636.76	3637.75	3626.78	1827.89	1813.89	G	189.12	171.11	172.10	95.06	2
34							L	132.10	114.09	115.07	66.55	1

Supp. Fig. 6C

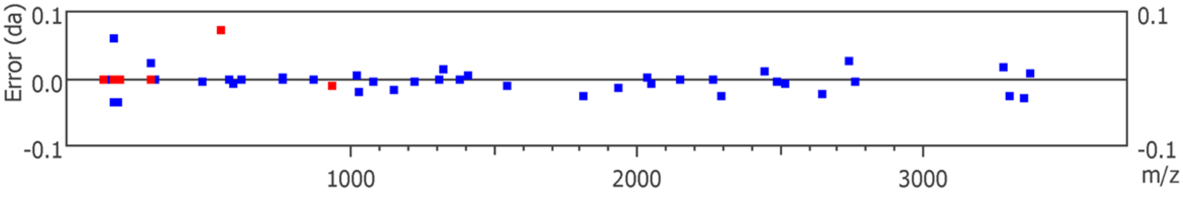

Supp. Fig. 6D

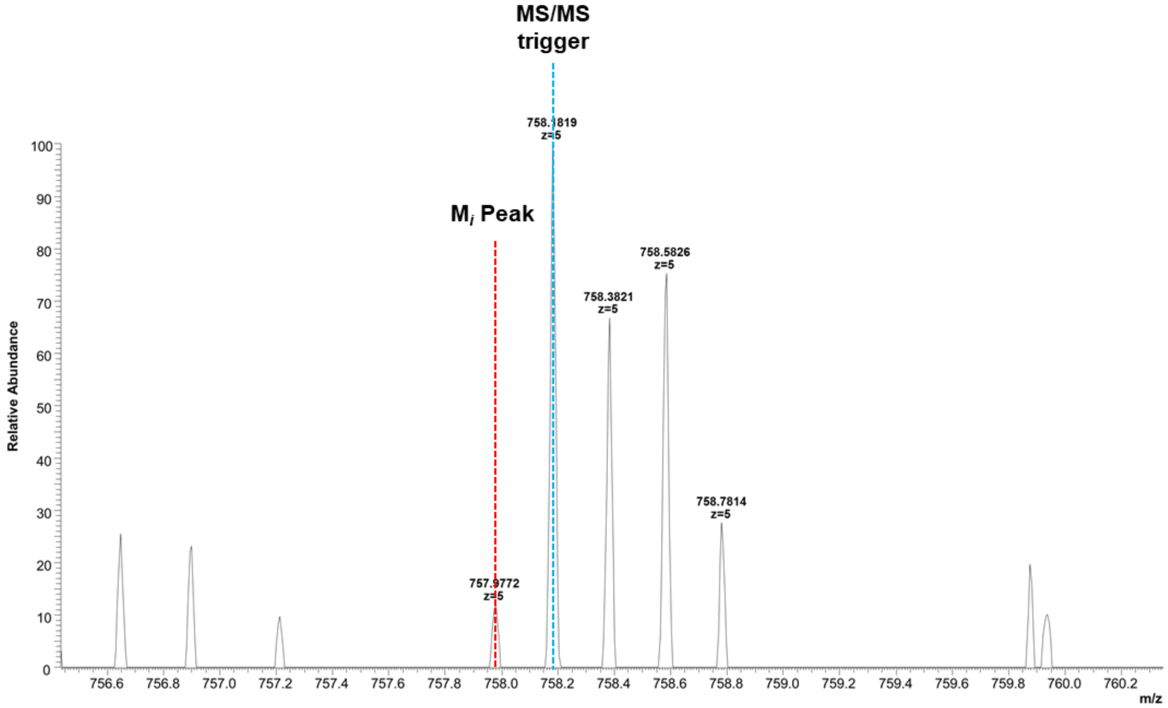

Supplementary Figure 6. Tandem mass spectrometry data for proteoform Aβ1-34. **A.** Annotated sequence Aβ1-34 showing sites of fragment ion cleavage (vertical lines), *b* ions (underlines), and *y* ions (overlines). **B.** Complete list of theoretical fragment ions for this proteoform, with observed *b* ions indicated in blue and observed *y* ions indicated in red. **C.** Error map of observed predicted mass/charge (*m/z*) ratio exported from PEAKS8. Blue indicates *b* ions, and red indicates *y* ions. **D.** Precursor ion isotopic envelope for this proteoform (-10logP: 61.66; RT: 51.60; Scan#7471; File: Pt6_F_SolAgg_tech-rep2). The monoisotopic peak (M_i) is delineated in the isotopic envelope with dashed *red* line, while the peak the MS/MS triggered on is demarked with the dashed *blue* line. The observed *m/z* was 757.9772 (theoretical *m/z* = 757.9792), which was -2.6 parts per million (ppm) error from the theoretical mass of Aβ1-34.

Supplementary Fig. 7A

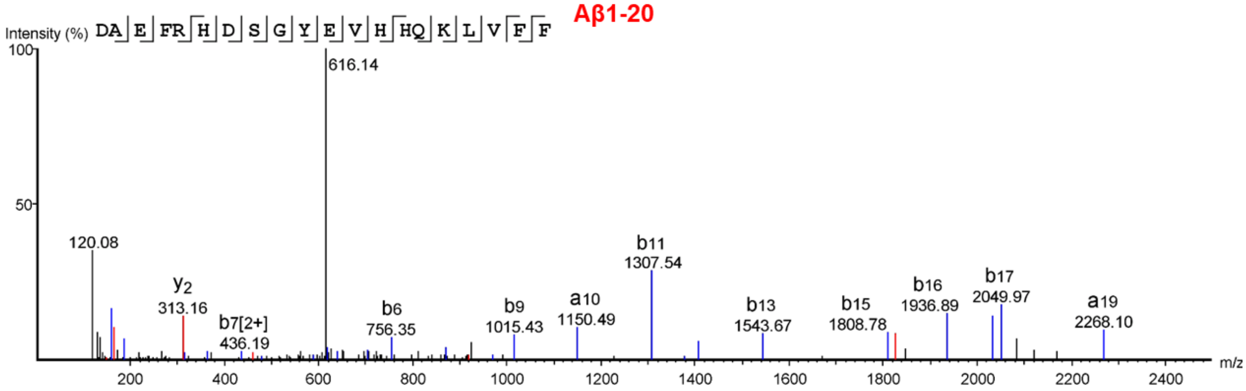

Supp. Fig. 7B

#	b	b-H2O	b-NH3	a	b (2+)	a (2+)	Seq	y	y-H2O	y-NH3	y (2+)	#
1	116.0348	98.0242	99.0078	88.0398	58.5174	44.5199	D					20
2	187.0724	169.0613	170.0449	159.0770	94.0359	80.0384	A	2346.1414	2328.1309	2329.1143	1173.5707	19
3	316.1132	298.1039	299.0875	288.1194	158.5572	144.5597	E	2275.1042	2257.0938	2258.0771	1138.0521	18
4	463.1829	445.1723	446.1559	435.1879	232.0914	218.0939	F	2146.0618	2128.0513	2129.0347	1073.5309	17
5	619.2917	601.2734	602.2570	591.2889	310.1420	296.1445	R	1998.9933	1980.9828	1981.9663	999.9966	16
6	756.3486	738.3323	739.3159	728.3479	378.6714	364.6780	H	1842.8921	1824.8816	1825.8185	921.9460	15
7	871.3677	853.3593	854.3428	843.3748	436.1906	422.1874	D	1705.8333	1687.8228	1688.8063	853.4166	14
8	958.4019	940.3913	941.3749	930.4069	479.7019	465.7034	S	1590.8063	1572.7958	1573.7793	795.9031	13
9	1015.4252	997.4127	998.3963	987.4283	508.2116	494.2141	G	1503.7743	1485.7638	1486.7473	752.3871	12
10	1178.4866	1160.4761	1161.4596	1150.4857	589.7446	575.7458	Y	1446.7528	1428.7423	1429.7258	723.8764	11
11	1307.5404	1289.5187	1290.5022	1279.5342	654.2646	640.2925	E	1283.6895	1265.6790	1266.6625	642.3447	10
12	1406.5870	1388.5872	1389.5707	1378.6154	703.8173	689.8013	V	1154.6469	1136.6364	1137.6199	577.8234	9
13	1543.6709	1525.6460	1526.6295	1515.6615	772.3282	758.3307	H	1055.5785	1037.5680	1038.5515	528.2892	8
14	1680.7155	1662.7050	1663.6885	1652.7205	840.8577	826.8602	H	918.5037	900.5090	901.4926	459.7666	7
15	1808.7792	1790.7635	1791.7471	1780.7791	904.8870	890.8895	Q	781.4606	763.4501	764.4337	391.2303	6
16	1936.8936	1918.8585	1919.8420	1908.8740	968.9484	954.9370	K	653.4020	635.3915	636.3751	327.2010	5
17	2049.9729	2031.9436	2032.9261	2021.9581	1025.4766	1011.4791	L	525.3071	507.2965	508.2801	263.1536	4
18	2149.0215	2131.0110	2131.9944	2121.0266	1075.0107	1061.0133	V	412.2231	394.2125	395.1961	206.6115	3
19	2296.0898	2278.0793	2279.0627	2268.1001	1148.5449	1134.5475	F	313.1556	295.1441	296.1277	157.0773	2
20							F	166.0865	148.0757	149.0597	83.5431	1

Supp. Fig. 7C

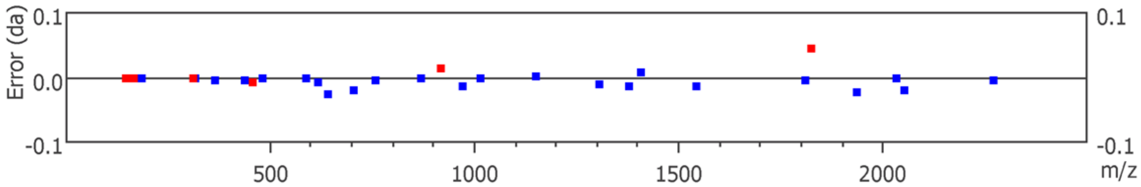

Supp. Fig. 7D

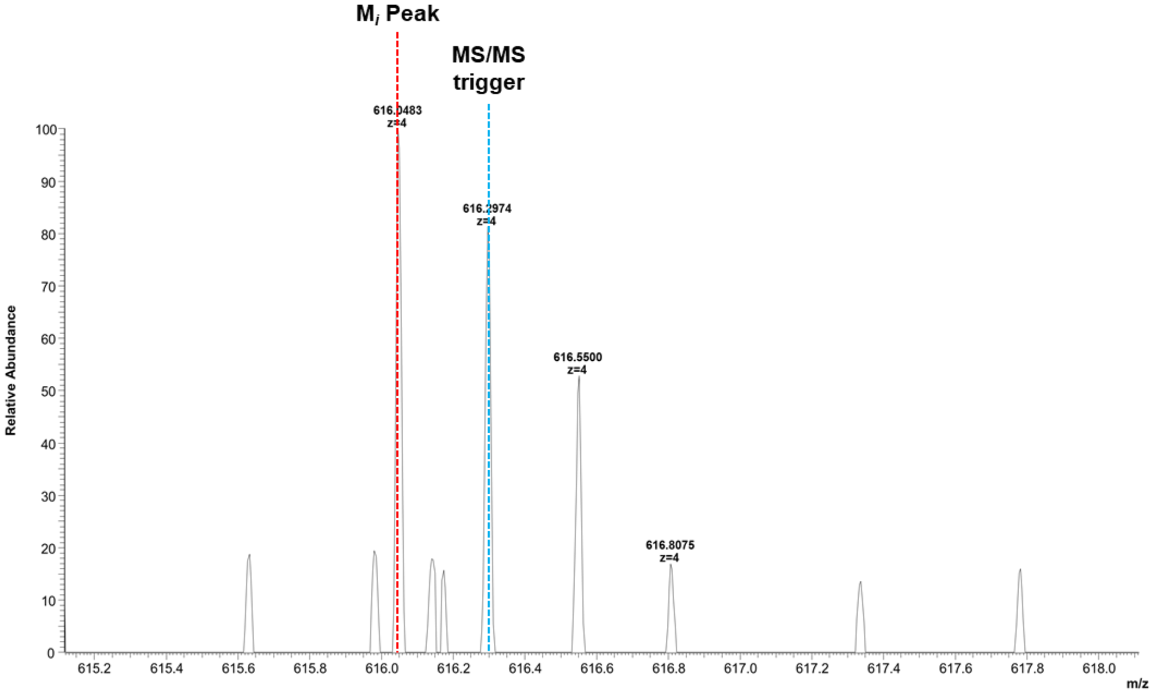

Supplementary Figure 7. Tandem mass spectrometry data for proteoform Aβ1-20. A. Annotated sequence Aβ1-20 showing sites of fragment ion cleavage (vertical lines), *b* ions (underlines), and *y* ions (overlines). **B.** Complete list of theoretical fragment ions for this proteoform, with observed *b* ions indicated in blue and observed *y* ions indicated in red. **C.** Error map of observed vs predicted mass/charge (*m/z*) ratio exported from PEAKS8. Blue indicates *b* ions, and red indicates *y* ions. **D.** Precursor ion isotopic envelope for this proteoform (-10logP: 47.74; RT: 41.26; Scan#6710; File: Pt4_F_SolAgg_tech-rep1). The monoisotopic peak (M_i) is delineated in the isotopic envelope with dashed *red* line, while the peak the MS/MS triggered on is demarked with the dashed *blue* line. The observed *m/z* was 616.0483 (theoretical *m/z* = 616.0475, which was 1.3 parts per million (ppm) error from the theoretical mass of Aβ1-20.

Supplementary Fig. 8A

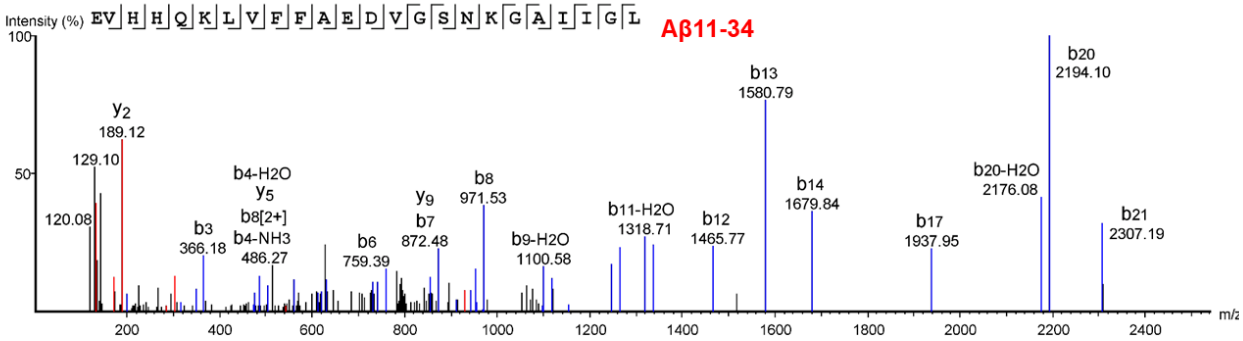

Supp. Fig. 8B

#	b	b-H2O	b-NH3	a	b (2+)	a (2+)	Seq	y	y-H2O	y-NH3	y (2+)	#
1	130.0504	112.0399	113.0234	102.0554	65.5252	51.5277	E					24
2	229.1188	211.1083	212.0918	201.1232	115.0594	101.0619	V	2479.3455	2461.3350	2462.3184	1240.1727	23
3	366.1772	348.1632	349.1508	338.1827	183.5889	169.5914	H	2380.2771	2362.2666	2363.2500	1190.6385	22
4	503.2364	485.2217	486.2740	475.2397	252.1183	238.1208	H	2243.2183	2225.2078	2226.1912	1122.1091	21
5	631.2896	613.2940	614.2682	603.3002	316.1128	302.2092	Q	2106.1592	2088.1487	2089.1321	1053.5796	20
6	759.3860	741.4060	742.3632	731.3740	380.1951	366.1772	K	1978.1007	1960.0902	1961.0737	989.5504	19
7	872.4783	854.4464	855.4622	844.4792	436.7371	422.7396	L	1850.0057	1831.9952	1832.9788	925.5029	18
8	971.5341	953.5253	954.5065	943.4843	486.2740	472.2755	V	1736.9216	1718.9111	1719.8947	868.9608	17
9	1118.5956	1100.5796	1101.5841	1090.6161	559.8118	545.8080	F	1637.8533	1619.8428	1620.8263	819.4266	16
10	1265.6890	1247.6577	1248.6525	1237.6844	633.3397	619.3413	F	1490.7849	1472.7744	1473.7579	745.8925	15
11	1336.7168	1318.7091	1319.6896	1308.7216	668.8583	654.8608	A	1343.7164	1325.7059	1326.6895	672.3582	14
12	1465.7665	1447.7487	1448.7322	1437.7642	733.3796	719.3821	E	1272.6793	1254.6688	1255.6523	636.8397	13
13	1580.7854	1562.7756	1563.7592	1552.7911	790.8931	776.8956	D	1143.6367	1125.6262	1126.6097	572.3184	12
14	1679.8428	1661.8440	1662.8275	1651.8595	840.4272	826.4297	V	1028.6099	1010.5992	1011.5828	514.8049	11
15	1736.8760	1718.8655	1719.8490	1708.8810	868.9380	854.9405	G	929.5370	911.5308	912.4695	465.2707	10
16	1823.9080	1805.8975	1806.8810	1795.9130	912.4695	898.4565	S	872.4783	854.4464	855.4622	436.7600	9
17	1937.9481	1919.9404	1920.9240	1909.9559	969.4780	955.4780	N	785.4879	767.4774	768.4609	393.2440	8
18	2066.0459	2048.0354	2049.0188	2038.0509	1033.5229	1019.5255	K	671.4450	653.4344	654.4180	336.2225	7
19	2123.0674	2105.0569	2106.0403	2095.0725	1062.0337	1048.0363	G	543.2750	525.3395	526.3231	272.1750	6
20	2194.1016	2176.0779	2177.0774	2166.1096	1097.5575	1083.5548	A	486.2740	468.3180	469.3016	243.6643	5
21	2307.1885	2289.1780	2290.1614	2279.1936	1154.0923	1140.0968	I	415.2915	397.2809	398.2645	208.1457	4
22	2420.2725	2402.2620	2403.2454	2392.2776	1210.6362	1196.6388	I	302.2092	284.1968	285.1358	151.6037	3
23	2477.2939	2459.2834	2460.2668	2449.2991	1239.1470	1225.1495	G	189.1241	171.1132	172.0964	95.0617	2
24							L	132.1023	114.0913	115.0749	66.5509	1

Supp. Fig. 8C

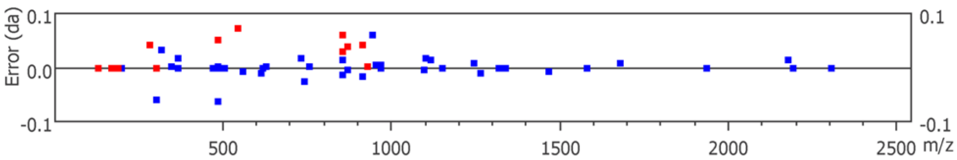

Supp. Fig. 8D

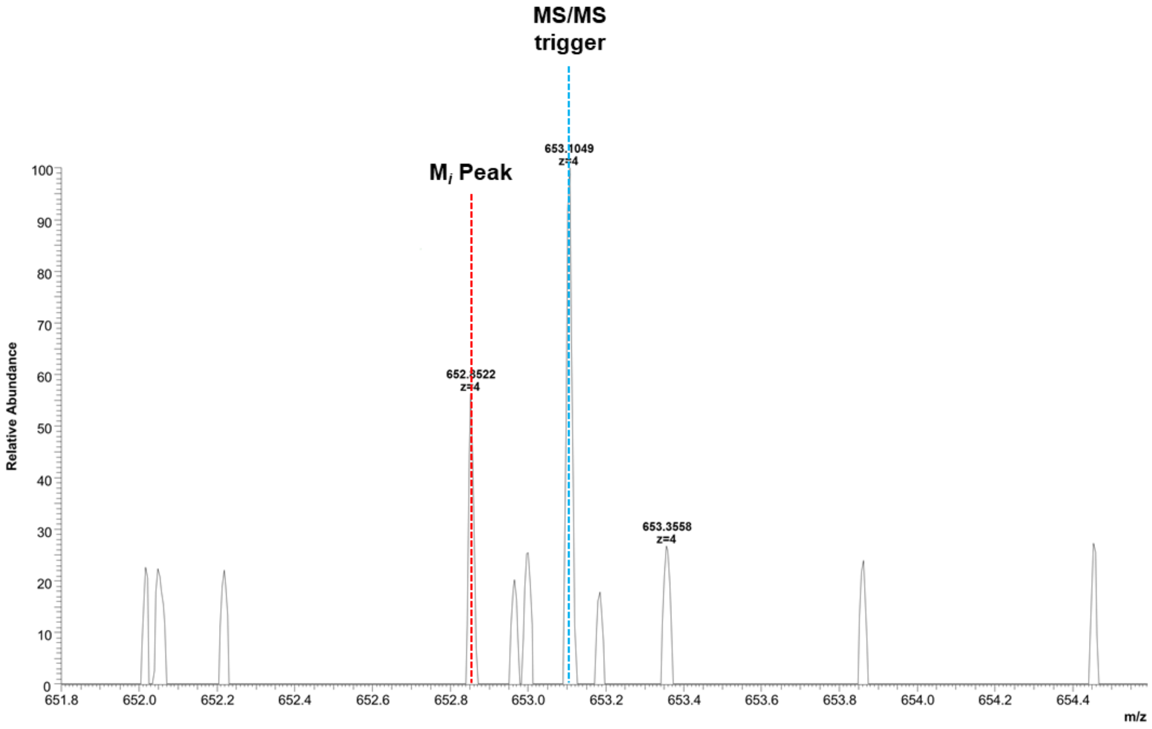

Supplementary Figure 8. Tandem mass spectrometry data for proteoform Aβ11-34. A. Annotated sequence Aβ11-34 showing sites of fragment ion cleavage (vertical lines), *b* ions (underlines), and *y* ions (overlines). **B.** Complete list of theoretical fragment ions for this proteoform, with observed *b* ions indicated in blue and observed *y* ions indicated in red. **C.** Error map of observed vs predicted mass/charge (*m/z*) ratio exported from PEAKS8. Blue indicates *b* ions, and red indicates *y* ions. **D.** Precursor ion isotopic envelope for this proteoform (-10logP: 59.56; RT: 52.52; Scan#8127; File: Pt5_F_SolAgg_tech-rep1). The monoisotopic peak (M_i) is delineated in the isotopic envelope with dashed *red* line, while the peak the MS/MS triggered on is demarked with the dashed *blue* line. The observed *m/z* was 652.8522 (theoretical *m/z* = 652.8525, which was -0.46 parts per million (ppm) error from the theoretical mass of Aβ11-34.

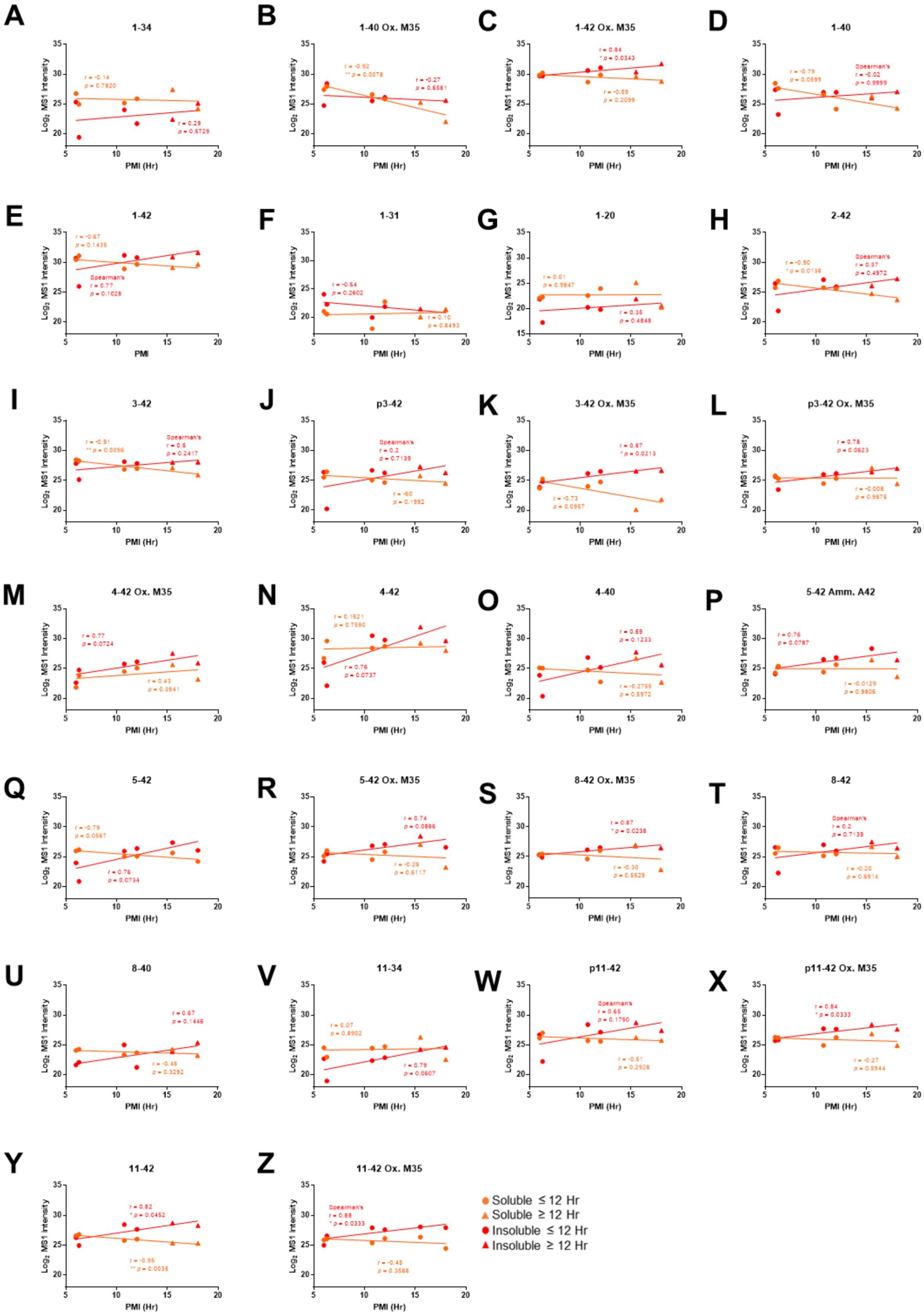
A 1-34
B 1-40 Ox. M35
C 1-42 Ox. M35
D 1-40
E 1-42
F 1-31
G 1-20
H 2-42
I 3-42
J p3-42
K 3-42 Ox. M35
L p3-42 Ox. M35
M 4-42 Ox. M35
N 4-42
O 4-40
P 5-42 Amm. A42
Q 5-42
R 5-42 Ox. M35
S 8-42 Ox. M35
T 8-42
U 8-40
V 11-34
W p11-42
X p11-42 Ox. M35
Y 11-42
Z 11-42 Ox. M35
Log2 MS1 Intensity
PMI (Hr)
Soluble ≤ 12 Hr
Soluble ≥ 12 Hr
Insoluble ≤ 12 Hr
Insoluble ≥ 12 Hr

Supplementary Figure 9: Soluble and more insoluble Aβ proteoform correlations with post-mortem interval. A. No significant correlation of soluble or more insoluble Aβ1-34 with PMI. **B**. High negative correlation of oxidized Aβ1-40 in the soluble fraction only with PMI. **C**. High positive correlation of oxidized Aβ1-42 in the more insoluble fraction only with PMI. **D-G**. No correlation ofAβ1-40, Aβ1-42, Aβ1-31, and Aβ1-20 in either the soluble or more insoluble fractions with PMI. **H.** High negative correlation of Aβ2-42 in the soluble fraction only with PMI. **I.** High negative correlation of Aβ3-42 in the soluble fraction only with PMI. **J.** No correlation with Aβp3-42 and PMI. **K.** High positive correlation of oxidized Aβ3-42 in the insoluble fraction only with PMI. **L-R.** No correlation of oxidized Aβp3-42, oxidized Aβ4-42, Aβ4-42, Aβ4-40, ammonium ion Aβ5-42, Aβ5-42, and oxidized Aβ5-42, in either the soluble or more insoluble fractions, with PMI. **S.** High positive correlation of oxidized Aβ8-42 in the more insoluble fraction only with PMI. **T-W.** No correlation of Aβ8-42, Aβ8-40, Aβ4-42, Aβ11-34, and Aβp11-42 in either the soluble or more insoluble fractions with PMI. **X.** High positive correlation of oxidized Aβp11-42 in the more insoluble fraction with PMI. **Y.** High positive correlation of Aβ11-42 in the more insoluble fraction and high negative correlation of Aβ11-42 in the soluble fraction with PMI. **Z.** High positive correlation of oxidized Aβ11-42 in the more insoluble fraction with PMI. All analyses were done using Pearsons's correlation coefficient unless otherwise stated. * $p \leq 0.05$; p ** ≤ 0.01.

Supplementary Table 1

Pt	Region	Tissue (g)	Total Protein Eluted (ng)	Total Aβ Eluted (ng)	Yield (Aβ as % of total protein)
1	P	1	1621.96	1.83	0.11
2	F	1	2558.94	3.14	0.13
3	F	2.56	6360.03	11.04	0.17
4	F	2.64	6319.65	7.49	0.12
4	P	2.76	5426.60	5.30	0.10
1	F	3.1	5627.76	21.13	0.38

Representative biochemical purifications of soluble high molecular weight Aβ aggregates by immuneprecipitation. NanoOrange was used to measure total protein and Aβ1-x ELISA to measure eluted Aβ. Pt, participant; P, parietal lobe; F, frontal lobe.

Supplementary Table 2

100k Insoluble HMW Aβ					
Pt	Input (ng)	Flowthrough (ng)	Wash (ng)	Elution (ng)	Salvage (ng)
2	6	6.84 ± 0.75	0.15 ± 0.03	0.15 ± 0.00	<LLoQ
3	6	4.90 ± 1.19	<LLoQ	0.07 ± 0.00	<LLoQ
3	6	4.29 ± 0.56	0.09 ± 0.00	0.08 ± 0.01	0.10 ± 0.00
4	6	6.93 ± 0.50	<LLoQ	<LLoQ	<LLoQ

Soluble HMW Aβ Aggregates					
Pt	Input (ng)	Flowthrough (ng)	Wash (ng)	Elution (ng)	Salvage (ng)
1	3	2.60 ± 0.82	<LLoQ	0.10 ± 0.02	<LLoQ
2	5	3.49 ± 0.91	<LLoQ	0.17 ± 0.00	<LLoQ

Aβ C_8 solid phase extraction separation characteristics. Quantitative tracking of total Aβ monomer equivalents during each step of the C_8 SPE process. Input, starting amount of Aβ placed in the C_8 SPE tip after conditioning and equilibration with 60% ACN/0.05% TFA and 0.05% TFA (*aq*), respectively; Flowthrough, unbound input material eluting through the tip; Wash, material eluting after wash steps in 10% ACN/10% TFA; Elution, material eluting after wash with 60% ACN/10% TFA; Salvage, material remaining in the sample tube salvaged with neat formic acid. SPE, solid phase extraction; Pt, participant; LLoQ, lower limit of quantitation.

SUPPLEMENTARY DISCUSSION

Summary

In summary, we have developed a method for further enrichment of Aβ from the other, as-yet-uncharacterized protein components of the soluble Aβ aggregates and a top-down (undigested) nLC-MS/MS method for the mass spectrometric characterization of Aβ proteoforms (**Fig. 1, Supp. Fig. 1-3, Supp. Table 1-2**). Applying this combined approach to soluble aggregates and insoluble material in brains of 6 cases with severe dementia and pathologically confirmed AD, we found diversity of Aβ peptides – 26 unique proteoforms total.

There are many unanswered questions arising from the data presented:

1. How do the observed truncations and PTMs affect AD pathology? The age of presentation of clinical dementia, rate of cognitive decline, and plaque growth/deposition?
2. If a large portion of Aβ peptide in both soluble and insoluble aggregates is modified (*e.g.*, truncated), how does this affect the binding and thus accuracy of PET-PIB and other amyloid-binding agents for imaging studies?
3. If Aβ is ~0.1% of total soluble aggregates based on Aβ1-x ELISA, what is the composition of the ~99.9% as-yet-uncharacterized protein components of the soluble aggregates?
4. How do the truncations and PTMs affect Aβ properties such as solubility, aggregation propensity, toxicity, interaction with 'binding partners' (*e.g.*, prion protein) [1], or recognition by the brain's immune response system, microglia?
5. Would we expect the same level of heterogeneity in the familial early-onset cases of AD?
6. What is the proteoform diversity in Aβ monomers and plaques, and which proteoforms are predominant in each fraction?
7. Is there any correlation between Apolipoprotein E genotypes and particular Aβ proteoforms?
8. Would any of these proteoforms be candidate pharmacodynamic markers for AD treatments?

We are in the early stages of characterizing Aβ proteoforms present in the soluble Aβ aggregates across the spectrum of AD progression using our top-down nLC-MS/MS approach followed by absolute quantitation of proteoforms that segregate AD from cognitively intact high pathology individuals. A full characterization of the Aβ proteoforms in a cross-sectional study of patient cohorts is beyond the scope of this communication.

Relationship to Previous Studies

PTMs are chemical modifications to proteins[2]. PTMs can result in changes in the physicochemical and biochemical properties of proteins, increasing the range of functional outcomes and providing an additional level of regulatory control [3,4]. The site localization, chemical nature of the PTM, and the microheterogeneity serve to precisely fine-tune the "information carrying capacity" of proteins within cellular networks [2,5]. One of the first described Aβ PTMs was pyro-glutamate at the third and eleventh amino acid residues of Aβ in

human AD brain[6]. However, analysis of Aβ proteoforms has been largely restricted to examination of plaques [6-10] or more recently CSF [11-13]. These studies, particularly plaque-based studies, came to varying conclusions as to which Aβ proteoform was most abundant. This lack of consensus is likely due to variations in methodology used to isolate Aβ and tools to identify the sequence structure. However, few studies have examined the PTMs present on soluble Aβ aggregates from either human or transgenic animal models. PTMs on soluble Aβ examined to-date include glutamine deamidation[14] and di-tyrosine crosslinking of synthetic Aβ[15], recombinantly expressed Aβ from CHO cells[16], and N-terminal extension[17] and O-linked glycosylation of CSF Aβ[18]. One study identified phosphorylation of serine 8 in transgenic mice and human AD tissue[19]. We found 26 unique Aβ proteoforms (**Fig. 2**) in severe AD brain. Of the 26 proteoforms identified none were phosphorylated[19], glycosylated[18], nitrated[20], or di-tyrosine linked[20]. Nor did we identify N-terminal extension, but rather extensive N- and C-terminal truncations. These negative results should be interpreted with caution, as we have not determined whether the methods we have used would have been sufficient for detection of these other PTMs or extended peptides. However, the clear trend of N-terminally truncated Aβ proteoforms having greater abundances in the more insoluble fraction relative to the soluble aggregates is in line with early work demonstrating that these types of peptides displayed enhanced aggregation via sedimentation analyses[21].

Advantages of our approach

The advantages of our approach include: *i*) the application of top-down (*i.e.*, intact, undigested) mass spectrometry to Aβ allows the unambiguous identification of the peptide and all its combinatorial truncations and PTMs. *ii*) The high resolution and high mass accuracy of the intact Aβ peptide and its fragment ions via tandem mass spectrometry (MS/MS) provide precise peptide sequencing information. *iii*) The discovery of both known and novel variations at proteome level. Other techniques like matrix-assisted laser desorption/ionization (MALDI) do not offer the same level of resolution and mass accuracy. In this study, it was the resolution and mass accuracy of the parent and fragment ions that allowed the unambiguous identification of peptide sequences and PTMs. Importantly, we employed MS/MS to fragment the peptides, producing fragment ions, for direct *de novo* sequencing, a technique not often applied in MALDI-based studies [11]. Finally, chromatographic separation of proteoforms before mass spectrometry analysis offers better sample separation, reducing sample complexity and improving resolution.

Limitations

While our approach offers numerous advantages, it is not without its limitations. There are numerous reports that detail types of proteoforms identified either by mass spectrometry or biochemistry[20] offering some insight as to what Aβ proteoforms we might expect to find in human AD brain. However, an extraction of soluble HMW Aβ aggregates as detailed in our previous work[22], followed by high-resolution mass spectrometry has never been performed before; thus, in practice we had no *a priori* knowledge. In our approach, we used a mass spectrometry data acquisition method commonly referred to as data-dependent acquisition (DDA). In DDA, MS/MS scans of a given proteoform are acquired once the proteoform has been detected in the preceding MS scan. A limitation to this approach is that MS/MS spectra acquired are done so in a stochastically sampled manner. In other words, the most abundant proteoforms are targeted for MS/MS before less abundant proteoforms. Further, since this in an

untargeted approach, due to the lack of *a priori* knowledge, not every MS/MS spectrum acquired is guaranteed to provide all the fragment ions needed to unambiguously determine a given sequence and/or PTM. An alternative approach in future studies would be data-independent acquisition (DIA), which does not require *a priori* knowledge like DDA, but instead fragments all precursors in a defined *m/z* window (MS/MS-based quantification[23]). The result is effectively a lower limit of detection (more sensitivity) compared to DDA (MS-based quantification)[23] due to increased selectivity[23,24]. However, DIA has been generally used and optimized for "bottom-up" (*i.e.*, proteolytically digested) samples[25] rather than "top-down" (undigested) samples. Coupled to these limitations, we used differential mass spectrometry (dMS) for proteoform quantitation in this study. Labels, both chemical and isotopic, commonly employed in bottom-up experiments are not readily applicable to top-down proteomics for quantitation. It is often difficult to chemically label intact proteins [26-28]. Isotopic labeling through stable isotopes, for example, is not guaranteed to incorporate into the entire protein and further can result in often difficult to interpret data due to overlapping isotopic envelopes and fragment ions [27]. Thus, we used dMS, which is reported to detect as low as 2-fold changes in protein or peptide expression between sample sets [29-31]. Until the issue of labeling intact proteins are addressed in the field, quantitation in top-down proteomics experiments will remain largely limited to dMS.

Ultimately, the question of whether the N- and C-terminal truncations and post-translational modifications of Aβ reflect findings relevant to living AD patients *vs*. post-mortem artefact remains unanswered despite our correlation analysis. Initial attempts to discern whether C-terminal truncations were an artefact of sample preparation by using ^{18}O water demonstrated incorporation of one to two heavy oxygen isotopes into the C-terminus of canonical Aβ1-42 (*data not shown*). Brain biopsy in living humans undergoing a craniotomy to relieve normal pressure hydrocephalus followed by immediate preparation by our protocol [22] would help address this question. The fact that several truncated Aβ proteoforms were found in the CSF of living human AD patients [12,13,17,32] reduces the likelihood that these specific proteoforms are the result of post-mortem or sample preparation artefacts. Yet, whether the heterogeneity of the proteoforms observed is a reflection of the 'age' of the peptide in brain – with more truncations and PTMs reflecting a longer lived peptide subjected to more processing – or if Aβ is rapidly processed after canonical beta- and gamma-secretase cleavage of amyloid precursor protein remains unresolved [33,34]. Future work utilizing either BACE1 inhibitors or general aspartic-acid protease inhibitors in the homogenization buffer could be a step forward in resolving some of aspects of this issue particularly with Aβp11-42 and Aβ11-42 (**Supp. Fig. 2** and **Supp. Fig. 9**). Further, while we take great care to remove all leptomeningeal, and intraparenchymal vessels to the fullest extent possible, it is impossible with our current method and that of others (to the best of our knowledge) to fully exclude all blood vessels (including capillaries). Given this limitation, we cannot exclude the possibility of contribution of Aβ proteoforms from vessel walls in our data. Aβ proteoforms from vessel walls may represent a different pool of proteoforms in AD brain.

Lastly, given the surprising level of heterogeneity observed in Aβ, it is now evident that our Aβ1-x ELISA is underestimating the total amount of Aβ, since the assay uses HJ3.4 as the detection antibody, which requires Aβ to have a free (unmodified) canonical Aβ N-terminus. As a result, an assessment of any Aβ proteoforms lost more readily than others throughout the purification process and C_8 micro-column procedure is lacking. In the absence of such an assay, future studies will employ an isotopic-labeled internal Aβ standard for data normalization.

Priorities for future research

1. Future studies will include a broader exploration of the spectrum of Aβ proteoforms present in the AD brain across disease severity – mild cognitive impairment, mild dementia, and severe dementia – compared to non-demented with high pathology controls and older healthy controls. Certain Aβ proteoforms may play an important role in the pathogenesis of AD. For example, if the predominant pathogenic Aβ proteoforms were C-terminally truncated, monoclonal antibodies targeting C-terminal truncated neo-epitopes of Aβ would have a higher chance of success in clinical trials compared to those targeting the N-terminus. Many monoclonal antibodies currently in clinical trials target the canonical N-terminus and have not shown success[35], yet, likewise, mid-domain monoclonal antibody Solanezumab has also recently shown disappointing results[36]. However, we cannot exclude the possibility that timing, dosage, or combination of therapeutics may still net an effect in reducing disease progression.

2. The characterization of Aβ proteoforms could lead to the targeted analysis of CSF or plasma in non-demented patient cohorts to identify which proteoforms may be a predictive marker for progression to clinical dementia.

3. Given the level of heterogeneity observed in Aβ, the development of an ELISA with capture and detection antibodies in the mid-domain would provide more accurate orthogonal measurements of total Aβ. However, the problems of cross reactivity with other amyloid precursor protein fragments and mid-domain steric hindrance would need to be addressed. If successful, this would likely improve consistency among reports of Aβ measurements using ELISAs to make therapeutic, prognostic, or diagnostic assessments.

Implications

The present findings provide an initial profile of Aβ proteoforms derived from soluble and more insoluble Aβ aggregates in human AD brain. Readily apparent from the data are the diversity of Aβ in human tissue. These data clearly demonstrate our dearth of knowledge in Aβ and the properties of these modified forms, which remain entirely unknown until further investigation.

Overall, this work *i*) developed an operational template for advancing our understanding of Aβ proteoform abundance and expression during disease progression, which will inform our therapeutic efforts. *ii*) Will facilitate direct comparison of human Aβ proteoforms to those found in transgenic animal models to determine which, if any, faithfully recapitulate those found in human AD [37,38]. Such an animal model would be the logical choice for novel AD therapeutic development, though it is possible no existing model accurately mimics the human disease, which would prompt the development of new model systems. *iii*) Enable direct comparison with Aβ proteoforms in the CSF to provide diagnostic and prognostic markers to distinguish AD patients from non-AD and monitor clinical trial treatment effects. Furthermore, the purification scheme and the top-down platform developed here may be used as a framework for the assessment of other human neurodegenerative diseases with hallmark proteinopathies, such as alpha-synuclein, prion protein, and superoxide dismutase 1 and therefore potential relevant therapeutic targets.

SUPPLEMENTARY REFERENCES

1 Laurén, J., Gimbel, D. A., Nygaard, H. B., Gilbert, J. W. & Strittmatter, S. M. Cellular prion protein mediates impairment of synaptic plasticity by amyloid-beta oligomers. *Nature* **457**, 1128-1132, doi:10.1038/nature07761 (2009).

2 Lichti, C. F. *et al.* in *Genomics and Proteomics for Clinical Discovery and Development Translational Bioinformatics 6* (ed G. Marko-Varga) Ch. 6, 101-136 (Springer Science+Business Media Dordrecht, 2014).

3 James, T. F. *et al.* The Nav1.2 channel is regulated by GSK3. *Biochim Biophys Acta* **1850**, 832-844, doi:10.1016/j.bbagen.2015.01.011 (2015).

4 Brooks, C. L. & Gu, W. Ubiquitination, phosphorylation and acetylation: the molecular basis for p53 regulation. *Curr Opin Cell Biol* **15**, 164-171 (2003).

5 Edwards, A. V., Edwards, G. J., Schwammle, V., Saxtorph, H. & Larsen, M. R. Spatial and temporal effects in protein post-translational modification distributions in the developing mouse brain. *J Proteome Res* **13**, 260-267, doi:10.1021/pr4002977 (2014).

6 Mori, H., Takio, K., Ogawara, M. & Selkoe, D. J. Mass spectrometry of purified amyloid beta protein in Alzheimer's disease. *J Biol Chem* **267**, 17082-17086 (1992).

7 Saido, T. C. *et al.* Dominant and differential deposition of distinct beta-amyloid peptide species, A beta N3(pE), in senile plaques. *Neuron* **14**, 457-466 (1995).

8 Kuo, Y. M., Emmerling, M. R., Woods, A. S., Cotter, R. J. & Roher, A. E. Isolation, chemical characterization, and quantitation of A beta 3-pyroglutamyl peptide from neuritic plaques and vascular amyloid deposits. *Biochem Biophys Res Commun* **237**, 188-191, doi:10.1006/bbrc.1997.7083 (1997).

9 Roher, A. E., Palmer, K. C., Yurewicz, E. C., Ball, M. J. & Greenberg, B. D. Morphological and biochemical analyses of amyloid plaque core proteins purified from Alzheimer disease brain tissue. *J Neurochem* **61**, 1916-1926 (1993).

10 Roher, A. E. *et al.* Structural alterations in the peptide backbone of beta-amyloid core protein may account for its deposition and stability in Alzheimer's disease. *J Biol Chem* **268**, 3072-3083 (1993).

11 Portelius, E., Westman-Brinkmalm, A., Zetterberg, H. & Blennow, K. Determination of beta-amyloid peptide signatures in cerebrospinal fluid using immunoprecipitation-mass spectrometry. *J Proteome Res* **5**, 1010-1016, doi:10.1021/pr050475v (2006).

12 Portelius, E. *et al.* Characterization of amyloid beta peptides in cerebrospinal fluid by an automated immunoprecipitation procedure followed by mass spectrometry. *J Proteome Res* **6**, 4433-4439, doi:10.1021/pr0703627 (2007).

13 Portelius, E. *et al.* Identification of novel APP/Abeta isoforms in human cerebrospinal fluid. *Neurodegener Dis* **6**, 87-94, doi:10.1159/000203774 (2009).

14 Schmid, A. W. *et al.* Tissue transglutaminase-mediated glutamine deamidation of beta-amyloid peptide increases peptide solubility, whereas enzymatic cross-linking and peptide fragmentation may serve as molecular triggers for rapid peptide aggregation. *J Biol Chem* **286**, 12172-12188, doi:10.1074/jbc.M110.176149 (2011).

15 Galeazzi, L., Ronchi, P., Franceschi, C. & Giunta, S. In vitro peroxidase oxidation induces stable dimers of beta-amyloid (1-42) through dityrosine bridge formation. *Amyloid* **6**, 7-13 (1999).

16 Tay, W. M. *et al.* A mass spectrometric approach for characterization of amyloid-β aggregates and identification of their post-translational modifications. *Biochemistry* **51**, 3759-3766, doi:10.1021/bi300316d (2012).
17 Brinkmalm, G. *et al.* An online nano-LC-ESI-FTICR-MS method for comprehensive characterization of endogenous fragments from amyloid-,âà√≠,Äö√¢¬ß and amyloid precursor protein in human and cat cerebrospinal fluid. *J Mass Spectrom* **47**, 591-603, doi:10.1002/jms.2987 (2012).
18 Halim, A. *et al.* Site-specific characterization of threonine, serine, and tyrosine glycosylations of amyloid precursor protein/amyloid beta-peptides in human cerebrospinal fluid. *Proc Natl Acad Sci U S A* **108**, 11848-11853, doi:10.1073/pnas.1102664108 (2011).
19 Kumar, S. *et al.* Extracellular phosphorylation of the amyloid β-peptide promotes formation of toxic aggregates during the pathogenesis of Alzheimer's disease. *EMBO J* **30**, 2255-2265, doi:10.1038/emboj.2011.138 (2011).
20 Kummer, M. P. & Heneka, M. T. Truncated and modified amyloid-beta species. *Alzheimers Res Ther* **6**, 28, doi:10.1186/alzrt258 (2014).
21 Pike, C. J., Overman, M. J. & Cotman, C. W. Amino-terminal deletions enhance aggregation of beta-amyloid peptides in vitro. *J Biol Chem* **270**, 23895-23898 (1995).
22 Esparza, T. J. *et al.* Purification and Quantitative Characterization of Soluble HMW Amyloid-beta from Alzheimer's disease Brain Lysates. *Manuscript in Preparation Scientific Reports* **6**, 38187, doi:10.1038/srep38187 (2016).
23 Egertson, J. D. *et al.* Multiplexed MS/MS for improved data-independent acquisition. *Nat Methods* **10**, 744-746, doi:10.1038/nmeth.2528 (2013).
24 Gillet, L. C. *et al.* Targeted data extraction of the MS/MS spectra generated by data-independent acquisition: a new concept for consistent and accurate proteome analysis. *Mol Cell Proteomics* **11**, O111 016717, doi:10.1074/mcp.O111.016717 (2012).
25 Egertson, J. D., MacLean, B., Johnson, R., Xuan, Y. & MacCoss, M. J. Multiplexed peptide analysis using data-independent acquisition and Skyline. *Nat Protoc* **10**, 887-903, doi:10.1038/nprot.2015.055 (2015).
26 Toby, T. K., Fornelli, L. & Kelleher, N. L. Progress in Top-Down Proteomics and the Analysis of Proteoforms. *Annu Rev Anal Chem (Palo Alto Calif)* **9**, 499-519, doi:10.1146/annurev-anchem-071015-041550 (2016).
27 Waanders, L. F., Hanke, S. & Mann, M. Top-down quantitation and characterization of SILAC-labeled proteins. *J Am Soc Mass Spectrom* **18**, 2058-2064, doi:10.1016/j.jasms.2007.09.001 (2007).
28 Cui, W., Rohrs, H. W. & Gross, M. L. Top-down mass spectrometry: recent developments, applications and perspectives. *Analyst* **136**, 3854-3864, doi:10.1039/c1an15286f (2011).
29 Wiener, M. C., Sachs, J. R., Deyanova, E. G. & Yates, N. A. Differential mass spectrometry: a label-free LC-MS method for finding significant differences in complex peptide and protein mixtures. *Anal Chem* **76**, 6085-6096, doi:10.1021/ac0493875 (2004).
30 Mazur, M. T. *et al.* Quantitative analysis of intact apolipoproteins in human HDL by top-down differential mass spectrometry. *Proc Natl Acad Sci U S A* **107**, 7728-7733, doi:10.1073/pnas.0910776107 (2010).
31 Ntai, I. *et al.* Applying label-free quantitation to top down proteomics. *Anal Chem* **86**, 4961-4968, doi:10.1021/ac500395k (2014).

32 Halim, A. *et al.* Site-specific characterization of threonine, serine, and tyrosine glycosylations of amyloid precursor protein/amyloid beta-peptides in human cerebrospinal fluid. *Proc Natl Acad Sci U S A* **108**, 11848-11853, doi:10.1073/pnas.1102664108 (2011).
33 Kim, I., Saito, T., Fujii, N. & Kanamoto, T. One-shot LC-MS/MS analysis of post-translational modifications including oxidation and deamidation of rat lens ,âà√≠¬¨¬±-and ,âà√≠,Äö√¢¬ß-crystallins induced by ,âà√≠,Äö√¢,Ä¢-irradiation. *Amino Acids*, doi:10.1007/s00726-016-2324-y (2016).
34 Sharma, K. K. & Santhoshkumar, P. Lens aging: effects of crystallins. *Biochim Biophys Acta* **1790**, 1095-1108, doi:10.1016/j.bbagen.2009.05.008 (2009).
35 Vandenberghe, R. *et al.* Bapineuzumab for mild to moderate Alzheimer's disease in two global, randomized, phase 3 trials. *Alzheimers Res Ther* **8**, 18, doi:10.1186/s13195-016-0189-7 (2016).
36 Doody, R. S. *et al.* Phase 3 trials of solanezumab for mild-to-moderate Alzheimer's disease. *N Engl J Med* **370**, 311-321, doi:10.1056/NEJMoa1312889 (2014).
37 Schieb, H. *et al.* Beta-amyloid peptide variants in brains and cerebrospinal fluid from amyloid precursor protein (APP) transgenic mice: comparison with human Alzheimer amyloid. *J Biol Chem* **286**, 33747-33758, doi:10.1074/jbc.M111.246561 (2011).
38 Kuo, Y. M. *et al.* Comparative analysis of amyloid-beta chemical structure and amyloid plaque morphology of transgenic mouse and Alzheimer's disease brains. *J Biol Chem* **276**, 12991-12998, doi:10.1074/jbc.M007859200 (2001).
